# Macropinocytic Uptake and pH‐Responsive Endolysosomal Processing Drive Sustained Chemotherapeutic Efficacy of High‐Load Core@Shell Nanocarriers in Colorectal Cancer

**DOI:** 10.1002/smsc.202500470

**Published:** 2025-12-15

**Authors:** Dolma Choezom, Silke Notter, Titus Griebel, Nathalia Ferreira, Johann Gruetz, Ajinkya Kulkarni, Matthias Schröter, Gražvydas Lukinavičius, Wiebke Möbius, Lena‐Christin Conradi, Claus Feldmann, Frauke Alves

**Affiliations:** ^1^ Translational Molecular Imaging, Clinic for Haematology and Medical Oncology University Medical Center Goettingen (UMG) 37075 Goettingen Germany; ^2^ Department of General, Visceral and Pediatric Surgery University Medical Center Goettingen (UMG) 37075 Goettingen Germany; ^3^ Translational Molecular Imaging Max‐Planck‐Institute for Multidisciplinary Sciences (MPI‐NAT) City Campus 37075 Goettingen Germany; ^4^ Institute of Inorganic Chemistry Karlsruhe Institute of Technology (KIT) 76131 Karlsruhe Germany; ^5^ Chromatin Labelling and Imaging Group Max‐Planck‐Institute for Multidisciplinary Sciences (MPI‐NAT) 37077 Goettingen Germany; ^6^ Department of Neurogenetics Max‐Planck‐Institute for Multidisciplinary Sciences (MPI‐NAT) 37075 Goettingen Germany; ^7^ Translational Molecular Imaging Institute for Clinical and Interventional Radiology University Medical Center Goettingen (UMG) 37075 Goettingen Germany

**Keywords:** chemotherapeutic cocktail, colon cancer, core@shell nanocarriers, drug delivery, intracellular trafficking, macropinocytosis

## Abstract

Poor tumor targeting, strong toxic side effects, and high drug resistance remain clinical challenges for conventional chemotherapy. Here, it is reported that drug‐cocktail core@shell nanocarriers are developed for the codelivery of lipophilic irinotecan (ITC) and the hydrophilic 5‐fluorouracil (5‐FU) metabolite (FdUMP), a commonly used combination in chemotherapy regimens for colorectal cancer. With a drug loading of 57% by mass, these nanocarriers achieve one of the highest reported drug payloads for a chemotherapeutic drug cocktail. Crucially, using a probe‐based imaging strategy with mechanistically responsive fluorescent reporters, we found that after slow uptake predominantly via macropinocytosis, the nanocarriers rapidly traffic to endolysosomal compartments, where the acidic environment triggers sustained drug release. In alignment with the slow uptake and trafficking behavior, these nanocarriers induce a delayed yet prolonged cytotoxic effect in colorectal cancer cells. These findings provide the first direct evidence linking slow uptake, intracellular trafficking, and progressive nuclear delivery of nanocarrier cargo to the delayed yet sustained cytotoxic response. Together, this work highlights both the therapeutic potential of these nanocarriers and the broad applicability of the probe‐based imaging approach to elucidate the mechanistic intracellular trafficking and nuclear delivery of different types of nanoparticles delivering cargoes beyond cancer chemotherapy in various cellular models.

## Graphical abstract

This study develops core@shell nanocarriers with an exceptionally high chemotherapy drug payload, exhibiting strong cytotoxic effects. Using this adaptable nanocarrier‐synthesis platform, herein, the cellular uptake pathway and intracellular journey of the nanocarriers are elucidated with different probe‐based imaging strategies.

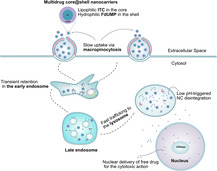

## 1 Introduction

Nanoparticle‐based drug‐delivery systems or nanocarriers have emerged as promising tools to enhance the therapeutic efficacy and safety profile of chemotherapeutic agents, holding the potential to overcome limitations of conventional treatments such as systemic toxicity and poor tumor selectivity.^[^ ^1^ ^]^ Among these, drug‐loaded nanocarriers, which are synthesized primarily from the drugs themselves, offer distinct advantages, including high drug payloads, minimal use of inert carrier materials, and improved pharmacokinetics.^[^ ^2^ ^]^ In our study, we designed nanocarriers using a core@shell architecture as previously described.^[^ ^3^ ^]^ The hydrophobic prodrug irinotecan (ITC) self‐assembles into the core, while the hydrophilic metabolite of 5‐fluorouracil (5‐FU), 5‐fluoro‐2′‐desoxyuridine‐5′‐monophosphate (FdUMP), forms the outer shell as ZrO(FdUMP). Both agents are chemotherapeutics frequently used together in combination regimens to treat colorectal cancer (CRC).^[^ ^4^ ^]^ This formulation strategy enables the codelivery of hydrophobic and hydrophilic chemotherapeutics in a single nanocarrier while minimizing the use of additional carrier material.

Despite significant progress in nanocarrier formulations to enhance drug delivery for a better treatment outcome, critical gaps remain in our understanding of how intracellular processing influences therapeutic efficacy. Specifically, the mechanisms governing cellular uptake, trafficking through endocytic compartments, and the kinetics of drug release from disintegrating nanocarriers are still largely unexplored. Yet, these intracellular processes are essential for maximizing therapeutic efficacy and minimizing off‐target toxicity. Endocytosis represents a major route of nanocarrier internalization, with different endocytic pathways exerting distinct effects on the intracellular fate and processing of nanocarriers.^[^ ^5^ ^]^ Furthermore, the membrane vesicle trafficking along the endosomal pathway culminating in the lysosome has gained particular attention due to its central role in nanocarrier degradation and in mediating their cellular responses.^[^ ^6^ ^]^ The lysosome, a membrane‐bound organelle with an acidic lumen enriched in hydrolytic enzymes, acts as a crucial compartment for the processing, degradation, and recycling of internalized materials.^[^ ^7^ ^]^

To address these questions, we employed a modular core@shell nanocarrier system coloaded with irinotecan and FdUMP to systematically investigate how intracellular uptake and trafficking influence drug release and cytotoxic response. Our adaptable platform allows the integration of functional dyes, enabling real‐time visualization of nanocarrier processing within cells. By correlating intracellular behavior with therapeutic outcome in colorectal cancer cells, this study aims to provide mechanistic insights that inform the rational design of more effective nanomedicine strategies.

## 2 Results

### 2.1 Synthesis and Nanocarrier Characterization

For a realistic in vivo application of drug nanocarriers, the drug load has to be as high as possible in order to limit the total number of nanocarriers as well as the liquid volume to reach clinically relevant drug concentrations. Moreover, the number and amount of additional auxiliary compounds, e.g., to make the nanocarriers chemically or colloidally stable, should be as low as possible in order not to decrease the amount of the active drug as well as to avoid toxic or allergic effects of these additional auxiliary compounds. Such considerations become even more important if a drug cocktail of two or more drugs is present in a single nanocarrier. If the total drug load is low anyway, the concentration of different drugs can become much too low in regard to clinically relevant concentrations. In this respect, we intend to realize nanocarriers with drug cocktails and a drug load as high as possible. Aiming at ITC and FdUMP as chemotherapeutic agents, we already presented nanocontainers with a drug load of 22 wt% ITC and 10 wt% FdUMP but with an additional 68 wt% of auxiliary compounds (e.g. surfactants).^[^ ^3^ ^]^ With this study, we have developed ITC/TocP@ZrO(FdUMP) core@shell nanocarriers with a significantly increased drug load of 28 wt% ITC and 29 wt% FdUMP, which represents the highest load of a drug cocktail reported for a single nanoparticle. Furthermore, the ITC:FdUMP ratio was improved to 1.0:1.8, which reflects the generally lower activity of FdUMP in comparison to ITC.

**Figure 1:**
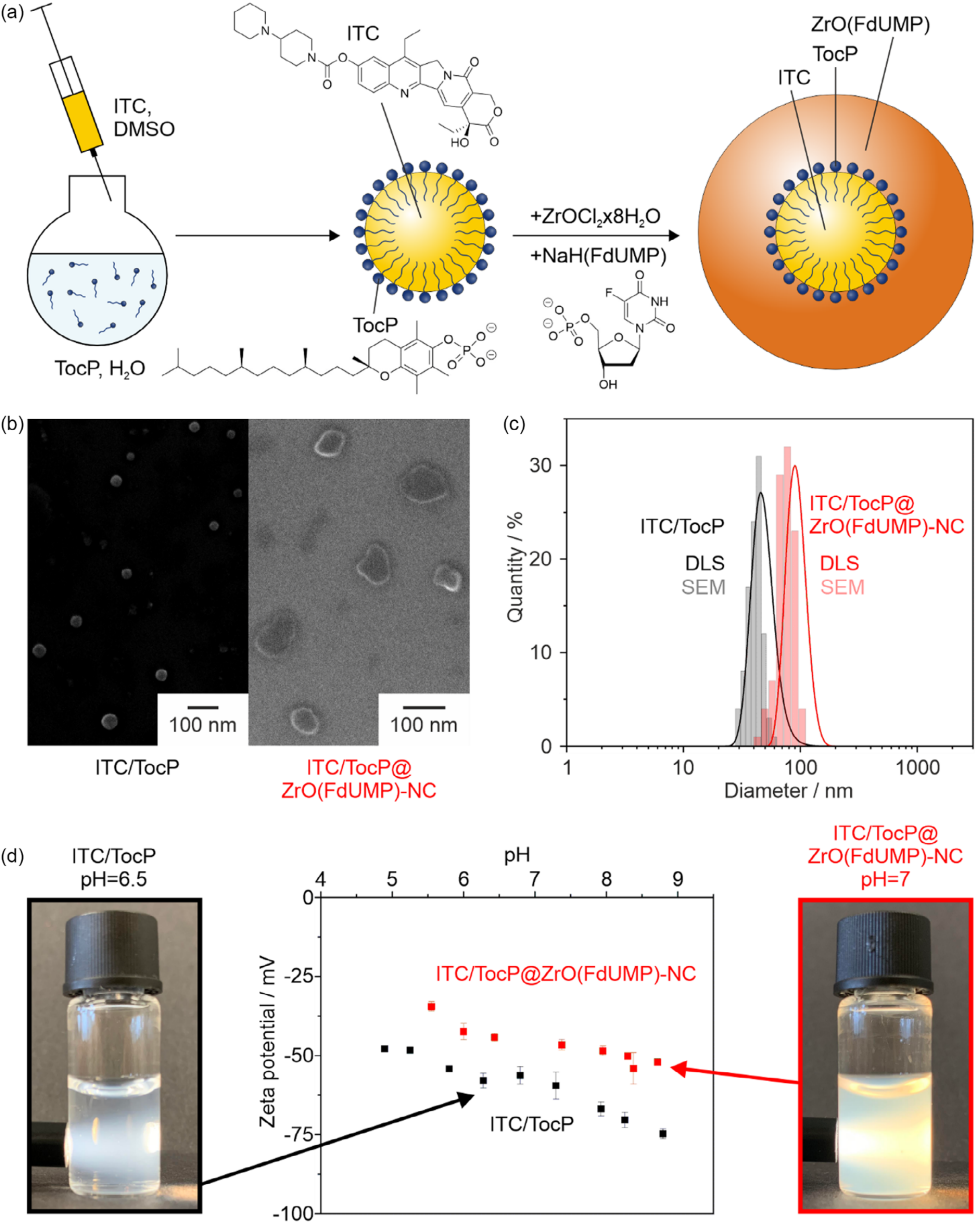
Synthesis and characterization of ITC/TocP@ZrO(FdUMP) core@shell nanocarriers: a) Schematic synthesis with solvent‐antisolvent approach to obtain the TocP‐stabilized ITC nanoparticles followed by the formation of the ZrO(FdUMP) shell around the ITC core. b) SEM images of TocP‐stabilized ITC nanoparticles and of ITC/TocP@ZrO(FdUMP) core@shell nanocarriers. c) Size distribution of TocP‐stabilized ITC nanoparticles and ITC/TocP@ZrO(FdUMP) core@shell nanocarriers according to DLS (in aqueous suspension) and SEM (statistical evaluation of 100 nanoparticles). d) Photos of suspension and zeta potential analysis.

ITC/TocP@ZrO(FdUMP) core@shell nanocarriers are prepared via an aqueous synthesis. First of all, nanoparticles of the lipophilic ITC (solubility in water: 0.11 mg mL^−1^) were realized via a so‐called solvent‐antisolvent approach, which we described previously (**Figure** **1**a).^[^ ^3^ ^]^ To stabilize the ITC nanoparticles and to avoid agglomeration, α‐tocopherolphosphate (TocP) was applied as a biocompatible surfactant (Figure 1a).^[^ ^8^ ^]^ Upon slow addition of [ZrO]^2+^ as an inorganic cation as well as of [FdUMP]^2−^ anions, a ZrO(FdUMP) shell containing the hydrophilic drug FdUMP (solubility in water: >50 mg mL^−1^) is formed and encapsulates the ITC core (Figure 1a). As a result, ITC/TocP@ZrO(FdUMP) core@shell nanocarriers were obtained with a total drug‐cocktail load of 57 wt% (thereof 28 wt% of lipophilic ITC and 29 wt% of hydrophilic FdUMP).

The course of the aforementioned synthesis can be followed by scanning electron microscopy (SEM), dynamic light scattering (DLS), as well as by zeta‐potential analysis. A statistical evaluation of 100 nanoparticles on SEM images reveals a spherical shape with a mean diameter of 41.2 ± 5.6 nm for the initial TocP‐stabilized ITC nanoparticles and a mean diameter of 75.4 ± 12.6 nm for the final ITC/TocP@ZrO(FdUMP) core@shell nanocarriers (Figure 1b,c). DLS of aqueous suspensions shows mean hydrodynamic diameters of 49.0 ± 11.1 nm of the TocP‐stabilized ITC nanoparticles and of 93.2 ± 16.3 nm of the ITC/TocP@ZrO(FdUMP) core@shell nanocarriers (Figure 1c). Both indicates an increase of the particle diameter after formation of the ZrO(FdUMP) shell, which exhibits a thickness of about 17 nm. Besides the increased particle diameter, the course of the synthesis can be specifically followed by the change of the surface charge as determined by zeta‐potential analysis. Thus, the initial TocP‐stabilized ITC nanoparticles exhibit a surface charge of –48 to –75 mV (at pH 5–9; Figure 1d). After formation of ZrO(FdUMP) and binding of [ZrO]^2+^ to the phosphate groups of TocP and FdUMP, the surface charge is decreased to −35 to −52 mV (at pH 5–9; Figure 1d). In both cases, the negative surface charging is causative for the good colloidal stability without the need of additional stabilizers or further surface functionalization. After purification by centrifugation and redispersion to remove excess starting materials and salts, the as‐prepared aqueous ITC/TocP@ZrO(FdUMP) nanocarrier suspensions are colloidally stable for several weeks.

To enable fluorescence tracking of the ITC/TocP@ZrO(FdUMP) core@shell nanocarriers in vitro and in vivo, red‐emitting fluorescent dyes can be added to the lipophilic ITC core as well as to the hydrophilic ZrO(FdUMP) shell. To this concern, small amounts of the lipophilic fluorescence red (FR, 1.9 × 10^−3^ mol%, *λ*(exc)_max_: 575 nm, *λ*(em)_max_: 615 nm) or of the hydrophilic phosphate‐functionalized dye DY‐647P1‐aadUTP (DUT647, 0.1 × 10^−3^ mol%, *λ*(exc)_max_: 647 nm, *λ*(em)_max_: 670 nm) were added in the synthesis together with the ITC or FdUMP solution (Figure S1, Supporting Information).

**Figure 2:**
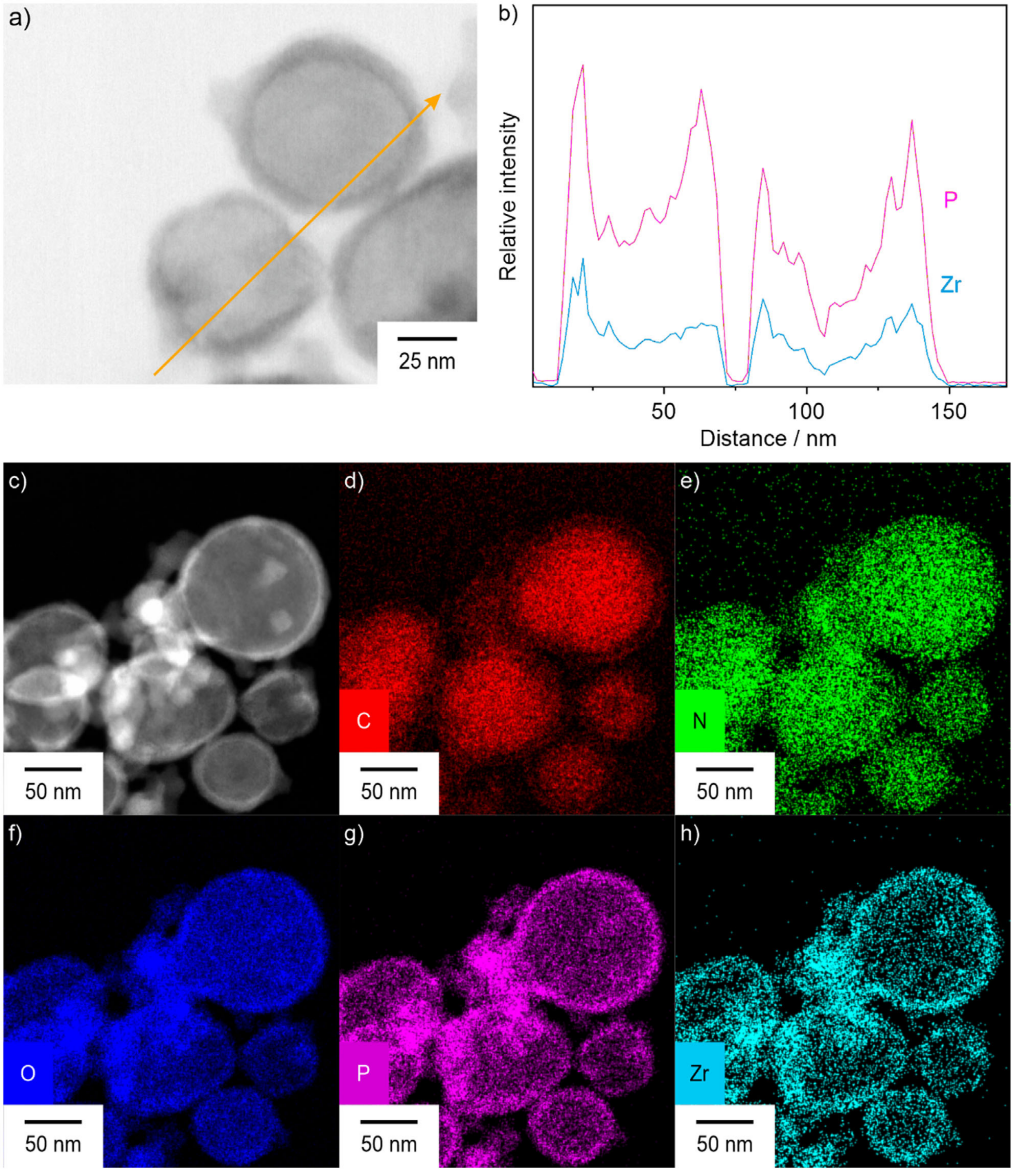
Size and structure of the ITC/TocP@ZrO(FdUMP) core@shell nanocarriers: a) STEM bright‐field image; b) EDXS linescan along the orange line in (a). c) STEM dark‐field image; d–h) EDXS element mappings of (d) C, (e) N, (f) O, (g) P, and (h) Zr.

Size and structure of the ITC/TocP@ZrO(FdUMP) core@shell nanocarriers were examined by scanning transmission electron microscopy (STEM) as well as by energy‐dispersive X‐ray spectroscopy (EDXS) (**Figure** **2**). STEM images of the ITC/TocP@ZrO(FdUMP) core@shell nanocarriers already indicate the presence of the shell based on the higher density and electron absorption of ZrO(FdUMP) (Figure 2a,c). EDXS linescans exhibit a characteristic U‐type concentration profile for Zr and P, indicating the presence of these elements only in the particle shell (Figure 2b). Moreover, the elemental maps show a homogeneous distribution of C, N, O (Figure 2d–f), but again a higher concentration of Zr and P in the particle shell (Figure 2g,h). Similar to SEM and DLS, the TocP‐stabilized ITC core exhibits a diameter of about 35 nm and the ZrO(FdUMP) shell a diameter of about 15 nm, resulting in a total diameter of about 65 nm.

Finally, the chemical composition of the ITC/TocP@ZrO(FdUMP) core@shell nanocarriers was analyzed by X‐ray powder diffraction (XRD), Fourier‐transform infrared (FT‐IR) spectroscopy, total organics combustion with thermogravimetry (TG), elemental analysis (EA) and photometry. Due to the high costs of NaH(FdUMP) (100 mg á 4,000 €), the analytical characterization was performed with ITC/TocP@ZrO(UMP) core@shell nanocarriers as Na_2_(UMP) is much cheaper (100 mg á 5 €). ZrO(FdUMP) and ZrO(UMP) differentiate only in the presence or absence of fluorine, which is highly relevant for the cytostatic activity but less relevant for the chemical composition. XRD indicates the nanocarriers to be amorphous (Figure S2, Supporting Information). This is to be expected for such material and synthesis temperature.^[^ ^9^ ^]^ Non‐crystallinity is also often beneficial due to a faster drug release compared to crystalline materials^[^ ^10^, ^11^ ^]^ FT‐IR spectra qualitatively confirm the presence of the constituents with *ν*(P=O) (1100, 990 cm^−1^) originating from TocP and UMP, *ν*(C=O) (1680 cm^−1^) originating from UMP, as well as pyridine‐related vibrations from ITC (*ν*(C=N), *ν*(C=C): 1620, 1600 cm^−1^) (Figure S3, Supporting Information). Total organics combustion with TG shows the release of water (up to 130 °C: 7 wt%) and the decomposition of ITC, TocP and UMP (up to 1200 °C: 68 wt% (Figure S4a, Supporting Information). The solid residue (25 wt%) was identified by XRD as ZrO_2_, ZrP_2_O_7_, and Zr_2_P_2_O_9_ (Figure S4b, Supporting Information). EA resulted in 43.7 wt% C, 4.7 wt% H, and 4.2 wt% N (see Supporting Information). These data are in good agreement with calculated values (38.5 wt% C, 4.6 wt% H, 4.4 wt% N, 73% total organic content), taking the amount and ratio of the starting materials into account (i.e., 3.75 mmol ITC, 3.61 mmol Na_2_(TocP), 10.8 mmol Na_2_(UMP), see Supporting Information). These values and the composition of the core@shell nanocarriers are also in agreement with a photometric analysis, which resulted in a load of 28 wt% ITC and 29 wt% UMP/FdUMP and an ITC: UMP/FdUMP ratio of 1:1.8 (Figure S5, Supporting Information).

In the following, the designation of the nanocarriers is simplified to ITC‐FdUMP‐NC in the case of ITC/TocP@ZrO(FdUMP) core@shell nanocarriers with two drugs. ITC‐NC refers to ITC/TocP@ZrO(UMP) core@shell nanocarriers with ITC only and FdUMP‐NC to TocP@ZrO(FdUMP) nanocarriers with FdUMP only. Furthermore, REF‐NC indicates drug‐free ZrO(TocP)/ZrO(UMP) reference nanocarriers (REF‐NC). REF‐ITC‐5FU indicates a reference solution with both dissolved drugs. REF‐ITC and REF‐5FU indicate solutions either containing only ITC or 5FU.

### 2.2 Nanocarriers Show Slow Uptake Kinetics When Compared to Transferrin

As a first step to dissect how these core@shell nanocarriers are internalized by cells, we evaluated their cellular uptake efficiency using DUT647‐labeled REF‐NC, which contained no active drug. The use of REF‐NC allowed for precise quantification of intracellular accumulation, eliminating potential confounding effects of the drugs.

**Figure 3:**
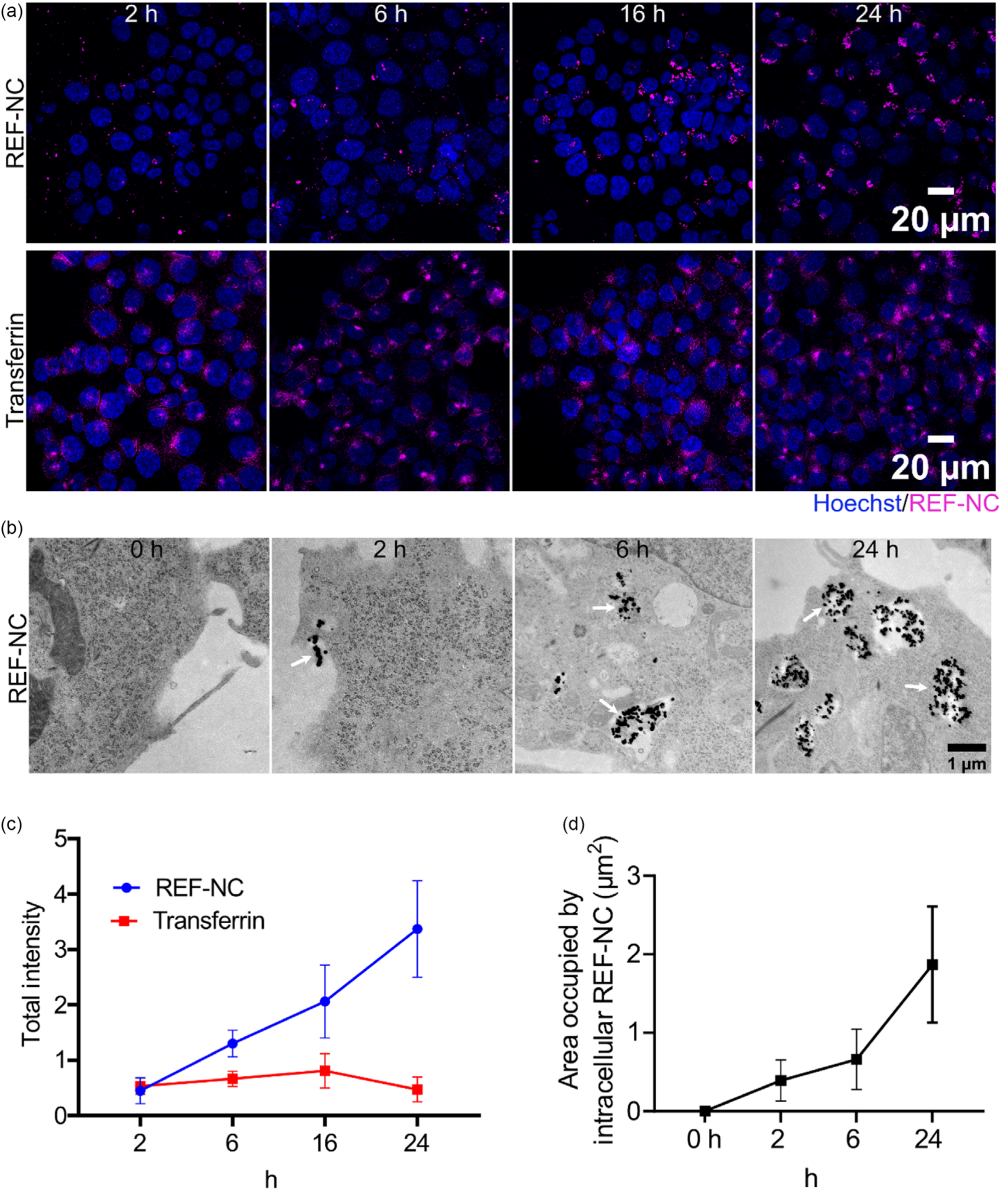
REF‐NC exhibit slow uptake kinetics when compared to transferrin: a) Representative confocal images of HCT116 cells treated with 60.53 μg mL^−1^ DUT647‐labeled REF‐NC (magenta) and transferrin conjugated to Alexa Fluor 647 (magenta) for 2, 6, 16, and 24 h. Nuclei were stained with Hoechst (blue). b) Representative electron microscopy images of HCT116 cells, untreated (0 h) or treated with 60.53 μg mL^−1^ REF‐NC for 2, 4, 6, or 24 h. c) Line graphs showing total intracellular REF‐NC intensity per cell, analyzed by CellProlifer segmentation with CellMask Green (not shown in images) and Hoechst staining from (A). d) Line graph showing quantification of EM images from (b) representing the area occupied by intracellular REF‐NC over time. Data in (c,d) are from two biological replicates.

To this end, we assessed the rate of internalization of REF‐NC by comparison to Alexa647‐labeled transferrin, a well‐characterized endocytic ligand that is known to be internalized via the clathrin‐mediated endocytic pathway (**Figure** **3**).^[^ ^12^, ^13^, ^14^ ^]^ The uptake kinetics, generated by measuring intracellular accumulation of the REF‐NC in comparison to Alexa647‐labeled transferrin at four different time points (2, 6, 16, 24 h) (Figure 3a), revealed distinct internalization rates for REF‐NC and transferrin (Figure 3c). Transferrin exhibited rapid internalization with intracellular accumulation visible at 2 h, which is consistent with its known efficient and fast uptake via clathrin‐mediated endocytosis^[^ ^13^ ^]^ (Figure 3a,c). In contrast, REF‐NC demonstrated a time‐dependent, slower uptake. The intracellular accumulation of REF‐NC increased gradually over time, reaching its maximum only after 24 h of exposure (Figure 3a,c).

To further validate the slow uptake kinetics of REF‐NC, we conducted high‐resolution transmission electron microscopy (TEM) to analyze both intracellular uptake and subcellular distribution of REF‐NC at distinct time points (2, 6, 24 h). Due to the incorporation of zirconium, a heavy element with strong electron absorbance, provides a reliable TEM signal for visualization of REF‐NC (Figure 3b). Similar to the observations with the confocal imaging (Figure 3a,c), quantifications of intracellular accumulation of REF‐NC over time on TEM images revealed a linear uptake profile, reaching its maximum at 24 h post incubation (Figure 3d).

Furthermore, TEM provided high‐resolution insights into the subcellular distribution of REF‐NC over time, revealing distinct trafficking patterns (Figure 3b). At 2 h, nanocarrier signals were predominantly detected within membrane‐bound organelles near the plasma membrane (PM), indicating early‐stage endocytosis with limited intracellular transport. By 6 h, REF‐NC‐containing vesicles were observed both near the nuclear region and at the cell periphery, suggesting active intracellular transport along the endocytic pathway. By 24 h, the majority of nanocarriers were localized within perinuclear compartments, indicating late endosomal/lysosomal accumulation (Figure 3b).

### 2.3 Core@Shell Nanocarriers are Predominantly Internalized Through Macropinocytosis

**Figure 4:**
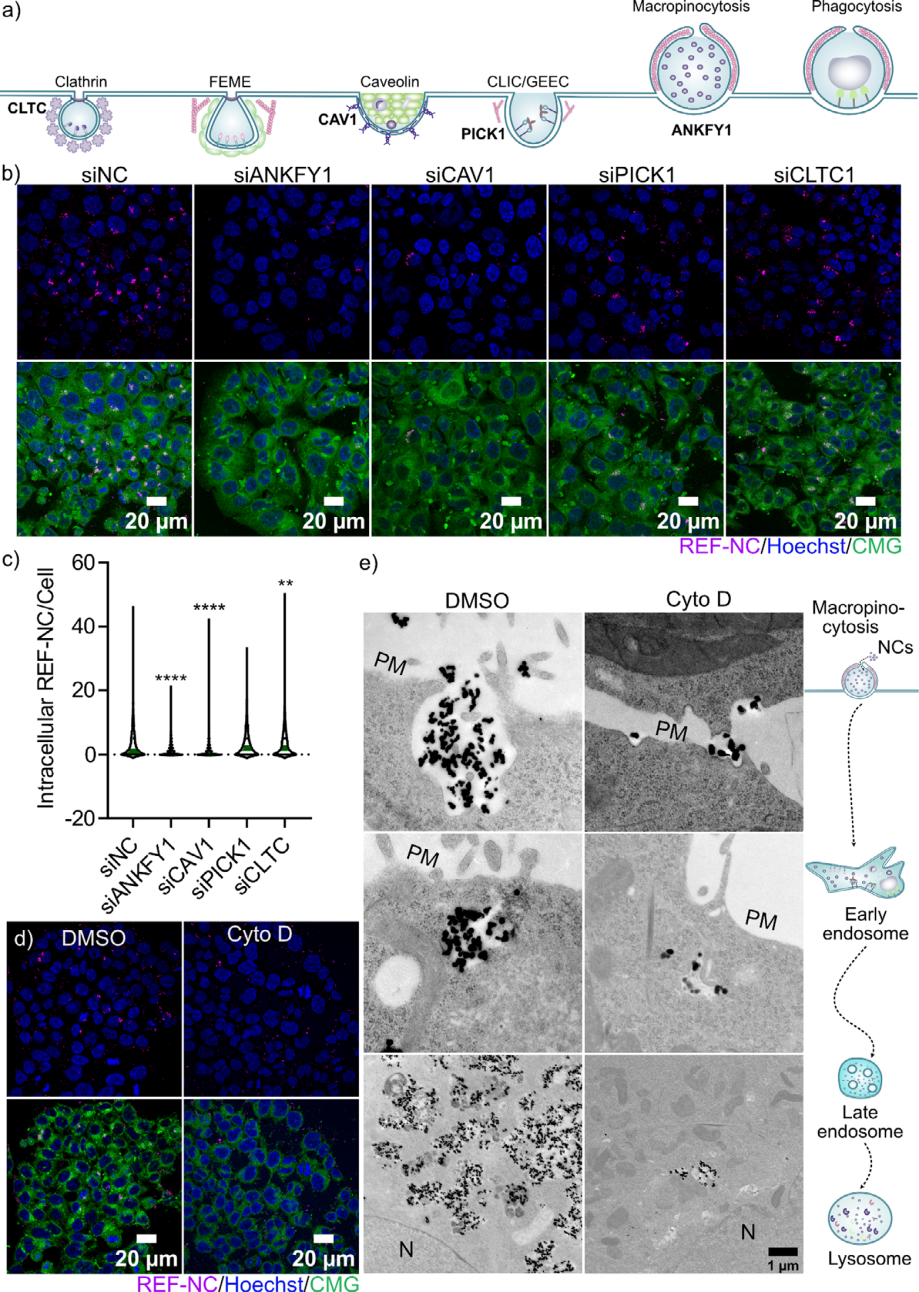
Core@shell nanocarriers are internalized through micropinocytosis. Scheme inspired from [5]: a) Scheme of endocytic pathways with the corresponding gene knockdowns (KDs) analyzed in this study. b) Representative confocal images of HCT116 cells treated with siRNA targeting the indicated genes for 48 h, followed by incubation with DUT647‐labeled REF‐NC for 6 h to assess uptake. The upper panel shows DUT647‐labeled REF‐NC (magenta) and nuclei stained with Hoechst (blue), while the lower panel includes an additional CellMask Green (CMG) stain to visualize cell membranes. c) Quantification of intracellular DUT647‐labeled REF‐NC per cell from (b), analyzed using CellProlifer with CMG and Hoechst staining for cell segmentation. Data are from three biological replicates; ^*^ *p* < 0.01, ^**^ *p* < 0.001, ^****^ *p* < 0.00001, (one‐way ANOVA with Tukey's multiple comparisons test). d) Representative confocal images of HCT116 cells pretreated with 20 μM Cytochalasin D (Cyto D) for 2 h before REF‐NC addition, followed by a 24 h incubation. e) Representative electron microscopy images of HCT116 cells pretreated with DMSO or cytochalasin D (Cyto D) for 2 h, followed by treatment with 60.53 μg mL^−1^ REF‐NC for 24 h. Left panel: Nanocarriers were observed in various intracellular trafficking compartments: near the plasma membrane (PM), undergoing internalization via macropinocytosis; within early endosomes at the cell periphery; and in late endosomes or lysosomes located close to the nucleus (N). Right panel: Cyto D treatment led to a marked reduction in nanocarrier uptake, particularly in compartments near the nucleus.

Nanocarriers primarily enter cells through endocytosis,^[^ ^5^ ^]^ and a detailed understanding of the specific internalization pathways involved in NCs can help optimize this potentially rate‐limiting step, guiding the design of nanocarriers to enhance uptake, particularly in tumor cells. To investigate the endocytic mechanisms responsible for core@shell NC uptake, we analyzed the uptake efficiency by measuring intracellular REF‐NC accumulation. We first analyzed the REF‐NC uptake efficiency in HCT116 cells following treatment with Dyngo and PITSTOP. Dyngo is a dynamin inhibitor that disrupts two dynamin‐dependent pathways: clathrin‐mediated endocytosis and fast endophilin‐mediated endocytosis (FEME).^[^ ^15^ ^]^ PITSTOP interferes with binding to the N‐terminal domain of clathrin, thereby specifically inhibiting clathrin‐mediated endocytosis^[^ ^16^ ^]^ (**Figure** **4**a). Surprisingly, we observed that the NC‐uptake efficiency remained unchanged upon treatment with both inhibitors, as both control and treated cells exhibited significant intracellular accumulation of REF‐NC comparable to their control treatments after overnight incubation (Figure S6a, Supporting Information). These results suggest that dynamin‐dependent pathways, including clathrin‐mediated endocytosis, are not the primary routes of NC internalization.

To further delineate the pathways involved in nanocarrier internalization, we performed gene knockdowns of key endocytic components for a specific pathway using siRNA, as illustrated in Figure 4a, and subsequently assessed uptake by measuring intracellular REF‐NC accumulation after 6 h of exposure (Figure 4b). Successful knockdown of all target genes was confirmed via qPCR (Figure S6b, Supporting Information).

Notably, knockdown of caveolin‐1 (CAV1), a key regulator of caveolin‐mediated endocytosis,^[^ ^17^ ^]^ and ANKFY1 (ankyrin repeat and FYVE domain‐containing 1), a mediator of micropinocytosis,^[^ ^18^ ^]^ significantly reduced REF‐NC uptake. In contrast, knockdown of clathrin heavy chain (CTLC), a major component of clathrin‐mediated endocytosis,^[^ ^19^ ^]^ led to an increased intracellular REF‐NC accumulation, suggesting an enhanced uptake. Meanwhile, knockdown of protein Interacting with C‐kinase 1 (PICK1), involved in FEME,^[^ ^20^ ^]^ had no detectable effect on REF‐NC uptake (Figure 4b,c). In alignment with reduced REF‐NC internalization upon ANKFY1 knockdown, we observed partial colocalization of REF‐NC with ANKFY1 proteins after 24 h (Figure S6c, Supporting Information, upper panel), but observed no colocalization between the CAV1 protein and REF‐NC at all the time points analyzed (Figure S6c, Supporting Information, lower panel*)*. These observations, therefore, indicate that macropinocytosis is one of the major internalization routes for NCs.

To validate the involvement of macropinocytosis in NC internalization, we analyzed the effects of cytochalasin D (Cyto D), a macropinocytosis inhibitor,^[^ ^21^ ^]^ on REF‐NC uptake efficiency first by confocal microscopy (Figure 4d). As expected, Cyto D treatment significantly reduced REF‐NC uptake after 18 h of exposure (Figure 4d and S7a, Supporting Information). Next, we further validated these findings using electron microscopy (EM), which revealed nanocarriers at various intracellular trafficking compartments under both dimethylsulfoxide (DMSO)‐ and cytochalasin D‐treated conditions after overnight exposure. Nanocarriers were observed at the cell surface PM, poised for internalization via membrane ruffling reminiscent of macropinocytosis, as well as within early endosomes and perinuclear endolysosomal compartments (Figure 4e, left panel). Notably, inhibition of macropinocytosis with Cyto D led to a marked reduction in nanocarrier uptake, particularly in compartments near the nucleus (Figure 4e, right panel). In agreement, the amount of REF‐NC per cell was significantly reduced upon micropinocytosis inhibition (Figure S7b,c, Supporting Information). Additionally, from the EM images (Figure 4e), the nanocarriers were observed to be engulfed as clusters of many individual nanocarriers by the invaginated macropinocytic membranes, suggesting that the collective size of these clusters may trigger micropinocytosis for their cellular uptake.

Therefore, based on all these findings, we propose that macropinocytosis is one of the major pathways mediating the uptake of core@shell nanocarriers.

### 2.4 Core@Shell Nanocarriers Undergo Intracellular Endosomal Trafficking

**Figure 5:**
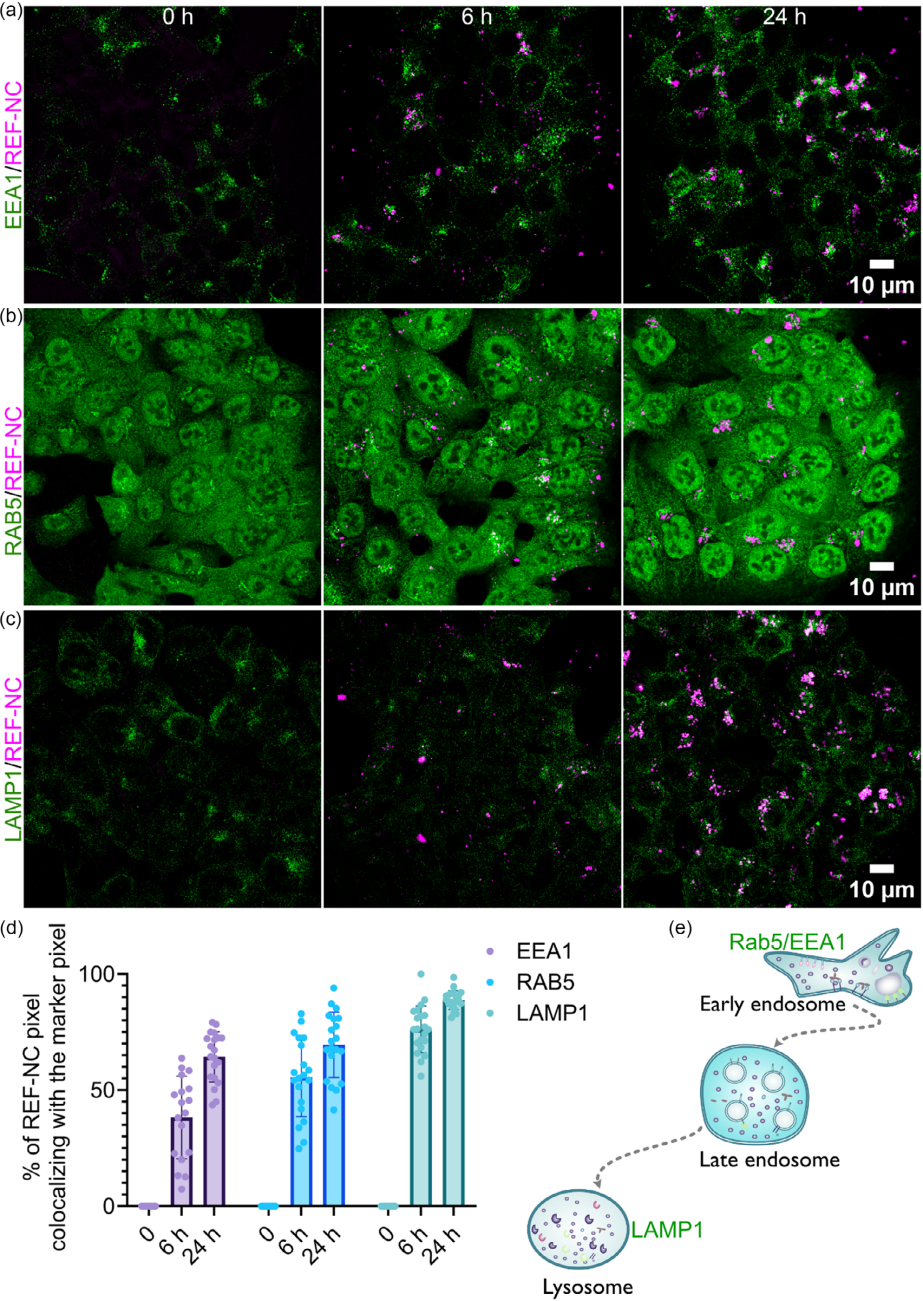
Core@shell nanocarriers undergo intracellular endosomal trafficking: a–c) Representative confocal microscopy images of HCT116 cells either untreated (0 h) or treated with 60.53 μg mL^−1^ DUT647‐labeled REF‐NC (margenta) for 6 or 24 h. Cells were immunostained for the endosomal markers EEA1 (a, green, early endosomes), RAB5 (b, green, early endosomes), and LAMP1 (c, green, lysosomes). d) Quantification of DUT647‐labeled REF‐NC colocalization with endosomal markers at different time points, expressed as the percentage of REF‐NC signal colocalizing with each marker. Data represent three independent experiments. e) Schematic representation of intracellular endosomal compartments, indicating key markers: early endosome (EEA1, RAB5), and lysosome (LAMP1).

Next, we investigated the intracellular trafficking pathways of core@shell nanocarriers after cellular uptake, examining how they navigate within the cell. To investigate REF‐NC intracellular trafficking, we performed confocal microscopy after costaining cells, exposed to REF‐NC at 6 h and 24 h, with various endosomal markers. Specifically, we labeled early endosomes with RAB5, a small GTPase regulating early endosomal trafficking, and its effector protein EEA1.^[^ ^22^ ^]^ Additionally, LAMP1, a lysosomal membrane protein,^[^ ^23^ ^]^ was used to assess lysosomal localization of REF‐NC after uptake (**Figure** **5**a–c,e).

At 6 h postincubation, REF‐NC exhibited partial colocalization with early endosomal markers RAB5 (≈55%) and EEA1 (≈35%), indicating its presence in early endosomal compartments (Figure 5a,b,d,e). By 24 h, colocalization with both markers increased to ≈70%, likely reflecting gradual but continuous uptake over time rather than prolonged retention, given the previously observed slow internalization kinetics (Figure 3). In parallel, a strong colocalization with the lysosomal marker LAMP1 (≈75%) was already evident at 6 h and further increased to ≈90% by 24 h (Figure 5c–e), confirming progressive trafficking of REF‐NC toward lysosomes.

Furthermore, we generated a stable HCT116 cell line expressing EGFP‐tagged RAB7A (EGFP‐RAB7A), a small GTPase associated with late endosomes, which plays a key role in the maturation and transport of endosomal vesicles.^[^ ^24^ ^]^ This allowed us to visualize and track the localization of REF‐NC in late endosomal compartments over time. Cells were incubated with REF‐NC for various time points and subsequently analyzed by confocal microscopy (Figure S8a, Supporting Information).

From 16 h postincubation onward, we began to observe clear localization of REF‐NC within RAB7A‐positive vesicles, indicating its progression from early to late endosomes. Interestingly, as the treatment duration increased, these RAB7A‐positive vesicles were visibly enlarged, suggesting a possible accumulation of nanocarriers and/or dynamic remodeling of the endosomal compartments in response to sustained nanocarrier exposure (Figure S8a, Supporting Information). These observations provide further evidence for the gradual and compartmentalized intracellular trafficking of REF‐NC through the endolysosomal pathway.

Overall, these findings suggest that REF‐NC follow a biphasic intracellular trafficking route. After gradual internalization, REF‐NC accumulate in early endosomes, as indicated by its increasing colocalization with RAB5 and EEA1 over time. However, a substantial fraction is rapidly transported to lysosomes, as evidenced by the high LAMP1 colocalization at early time points (Figure 5e).

### 2.5 Core@Shell Nanocarriers Undergo Endolysosomal Processing in a pH‐Dependent Manner for Cargo Release

After confirming that core@shell nanocarriers traffic through the endosomal pathway and reach the lysosome, we next investigated their degradation and drug release dynamics within endolysosomal compartments. Given that late endosomes and the lysosomes provide an acidic environment enriched with hydrolytic enzymes, we hypothesized that the nanocarriers could disintegrate within these compartments, thereby triggering drug release.

**Figure 6:**
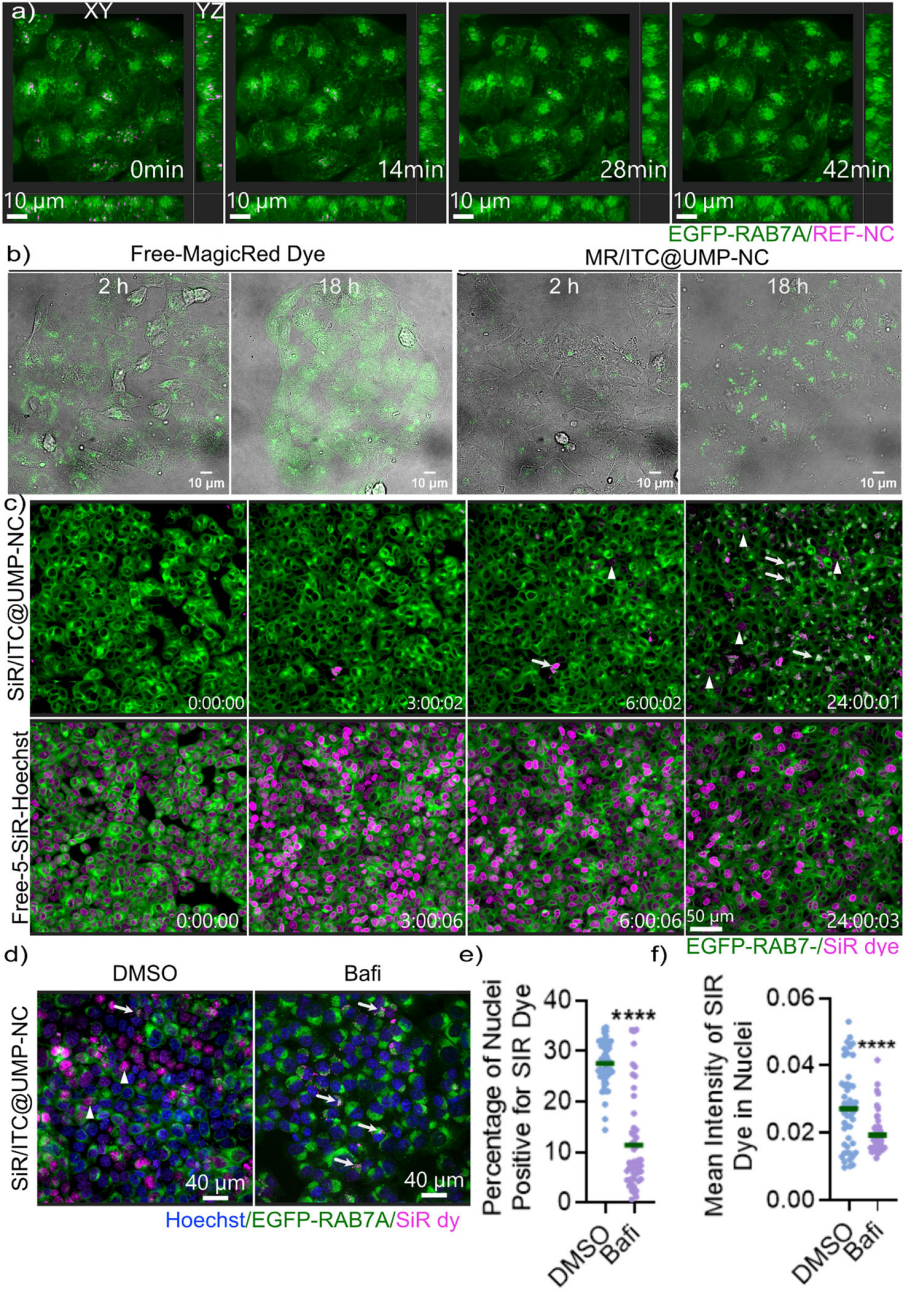
Core@shell nanocarriers undergo endosomal trafficking and lysosomal degradation for sustained intracellular drug release. a) Representative spinning‐disk microscopy image of live EGFP‐RAB7A‐expressing HCT116 cells (green) treated overnight with DUT647‐labeled REF‐NC. After washing, imaging was performed for 42 min. REF‐NCs localize to Rab7A‐positive vesicles, with fluorescence gradually decreasing over time, indicating nanocarrier degradation. b) Confocal microscopy images of HCT116 cells treated with 88 μg mL^−1^ of MR/ITC‐UMP‐NC or the equivalent amount of free MagicRed dye for 2 and 18 h. c) Representative spinning disk microscopy images of live EGFP‐RAB7A‐expressing HCT116 cells (green) treated before 0 h with either free 5‐SiR‐Hoechst dye or with core@shell NC encapsulating 5‐SiR‐Hoechst dye (SiR/ITC‐UMP‐NC), followed by imaging up to 24 h. Triangle signs indicate 5‐SiR‐Hoechst nuclear staining and arrows indicate endosomal 5‐SiR‐Hoechst signal. d) Representative spinning disk confocal microscopy images of EGFP‐RAB7A–expressing HCT116 cells (green) exposed to 75 μg mL^−1^ of SIR/ITC‐UMP‐NC for 6 h, followed by extensive washing to remove non‐internalized nanocarriers. Cells were then treated with 200 nM bafilomycin A1 (Bafi) for 18 h to inhibit endolysosomal acidification before fixation and imaging. White triangle signs indicate 5‐SiR‐Hoechst nuclear staining and white arrows indicate endosomal 5‐SiR‐Hoechst signals. e) Quantification of the percentage of nuclei (total nuclei segmented using Hoechst staining) positive for 5‐SiR‐Hoechst dye per image between DMSO and bafilomycin A1‐treated cells. f) Mean intensity of 5‐SiR‐Hoechst dye within nuclei, as segmented in (e), that overlaps with Hoechst staining. Data presented in (e,f) obtained from two biological replicates; ^****^ *p* < 0.0001; unpaired two‐tailed student's *t*‐test.

To test this, we performed live‐cell imaging with HCT116 cells stably expressing EGFP‐RAB7A. The snapshots of live‐cell imaging (**Figure** **6**a, Supporting Information Video) at various time points (14, 28, 42 min) revealed that REF‐NCs accumulate in Rab7A‐positive vesicles, where their fluorescence signal progressively diminishes over time, suggesting nanocarrier degradation (Figure 6a, Supporting Information Video).

Furthermore, to directly show nanocarrier degradation in the lysosome, we incorporated MagicRed Dye (MR), a fluorogenic lysosomal substrate,^[^ ^25^ ^]^ into the nanocarrier core along with the drug ITC (MR/ITC‐UMP‐NC). MR remains non‐fluorescent until cleaved by cathepsin L, a protease that is present predominantly in the lysosome. And therefore, freely dissolved MR is used as an established marker to assess lysosomal enzymatic activity.^[^ ^26^ ^]^ To prove nanocarrier trafficking and processing in the lysosome, we exposed cells to both free‐MR Dye and MR/ITC‐UMP‐NC at 2 and 18 h and observed MR fluorescence by confocal microscopy (Figure 6b). After 2 h of exposure, free MR dye produced a diffuse yet concentrated signal around the perinuclear area, whereas MR/ITC@UMP‐NC showed a weak but distinct fluorescence, exclusively localized in the perinuclear region. By 18 h, the free dye exhibited a highly diffused signal alongside perinuclear accumulation. In contrast, MR/ITC‐UMP‐NC displayed a strong punctate fluorescence pattern (Figure 6b), closely resembling REF‐NC localization (Figure 3, 4, 5). The progressive increase in punctate fluorescence with MR/ITC‐UMP‐NC indicates continual cellular uptake as well as trafficking and processing of the nanocarriers in the lysosomes, where they disintegrate and enable MR fluorescence activation by cathepsin L.

After confirming the degradation of the core@shell nanocarriers in lysosomes using MR dye, we next investigated the kinetics of drug release, particularly its translocation to the nucleus. Since many anticancer agents exert their effects in the nucleus, we used 5‐SiR‐Hoechst, a DNA‐binding dye,^[^ ^27^ ^]^ as a model cargo to assess nuclear delivery over time. To achieve this, 5‐SiR‐Hoechst was co‐encapsulated with ITC in the nanocarrier core (SiR/ITC‐UMP‐NC), and its nuclear staining was compared to that of free 5‐SiR‐Hoechst dye at various time points using live‐cell imaging in EGFP‐RAB7A‐expressing cells (Figure 6c, Supplementary video). While the free 5‐SiR‐Hoechst dye accumulated in the nucleus immediately after addition (0 h), reaching peak intensity within 3 to 6 h before declining at 24 h, the SiR/ITC‐UMP‐NC displayed a markedly delayed nuclear staining pattern. No detectable DNA signal was observed at 0 h and 3 h, a weak signal appeared in some cells at 6 h, and nuclear staining peaked only after 24 h. As expected, unlike the free 5‐SiR‐Hoechst dye, which showed a clear nuclear signal immediately after adding, SiR/ITC‐UMP‐NC showed colocalization with EGF‐RAB7A as early as 6 h, which was further increased by 24 h, confirming its trafficking through the endosomal pathway. Interestingly, a significant portion of NC remained accumulated in the late endosomes even after 24 h, confirming a slow and controlled dye release from the late endosomes.

To investigate whether our nanocarriers disintegrate in the acidic environment of lysosomes, we used bafilomycin A1 (Bafi), a potent V‐ATPase inhibitor resulting in reduced endolysosomal acidification.^[^ ^28^ ^]^ V‐ATPases are proton pumps located on acidic cellular membranes that actively pump protons into the lumen to maintain low pH.^[^ ^29^ ^]^ We hypothesized that the nanocarrier disintegration depends on this acidic milieu. To test this, we first allowed SiR/ITC‐UMP‐NC to be taken up by the cells for 6 h. After thoroughly washing out non‐internalized nanocarriers, we treated the cells with bafilomycin A1 for 18 h and assessed nuclear 5‐SiR‐Hoechst staining as a readout of drug release (Figure 6d). As expected, inhibition of lysosomal acidification by bafilomycin A1 significantly reduced both the percentage of nuclei positive for 5‐SiR‐Hoechst staining and the mean nuclear intensity of the dye (Figure 6d–f). Furthermore, upon lysosomal inhibition with bafilomycin A1, SiR/ITC‐UMP‐NC are more accumulated in the Rab7A+ endosomal compartments when compared to the control cells (Figure 6d). These results indicate that the nanocarrier disintegration within lysosomes indeed requires an acidic environment. Consistent with this, a reduction in endolysosomal acidification upon bafilomycin A1 treatment was confirmed by diminished LysoTracker staining (Figure S8b, Supporting Information).

Overall, we show that core@shell nanocarriers traffic through the endosomal pathway and accumulate in lysosomes, where their degradation enables gradual cargo release over time, followed by nuclear delivery. Compared to free 5‐SiR‐Hoechst dye, SiR/ITC‐UMP‐NC treatment resulted in delayed nuclear staining, consistent with controlled intracellular delivery. Inhibition of lysosomal acidification by bafilomycin A1 significantly impaired the nuclear accumulation of released 5‐SiR‐Hoechst, confirming that nanocarrier disintegration requires an acidic environment.

### 2.6 Comparative Efficacy of Core@Shell Nanocarriers and Free Drugs in Colorectal Cancer and Normal Retinal Epithelial Cells

After establishing the internalization, endosomal trafficking, and lysosomal degradation of the core@shell nanocarriers for drug release, we next investigated how these properties influence the therapeutic efficacy of ITC‐ and FdUMP‐loaded core@shell nanocarriers. The observed slow uptake kinetics and delayed nuclear delivery suggest a more sustained intracellular drug retention, potentially leading to a prolonged cytotoxic effect compared to free drugs. To evaluate this, we performed in vitro studies to compare the cytotoxic effects of nanocarrier‐delivered chemotherapeutics (ITC‐FdUMP‐NC, ITC‐NC, FdUMP‐NC) with their free‐drug counterparts.

**Figure 7:**
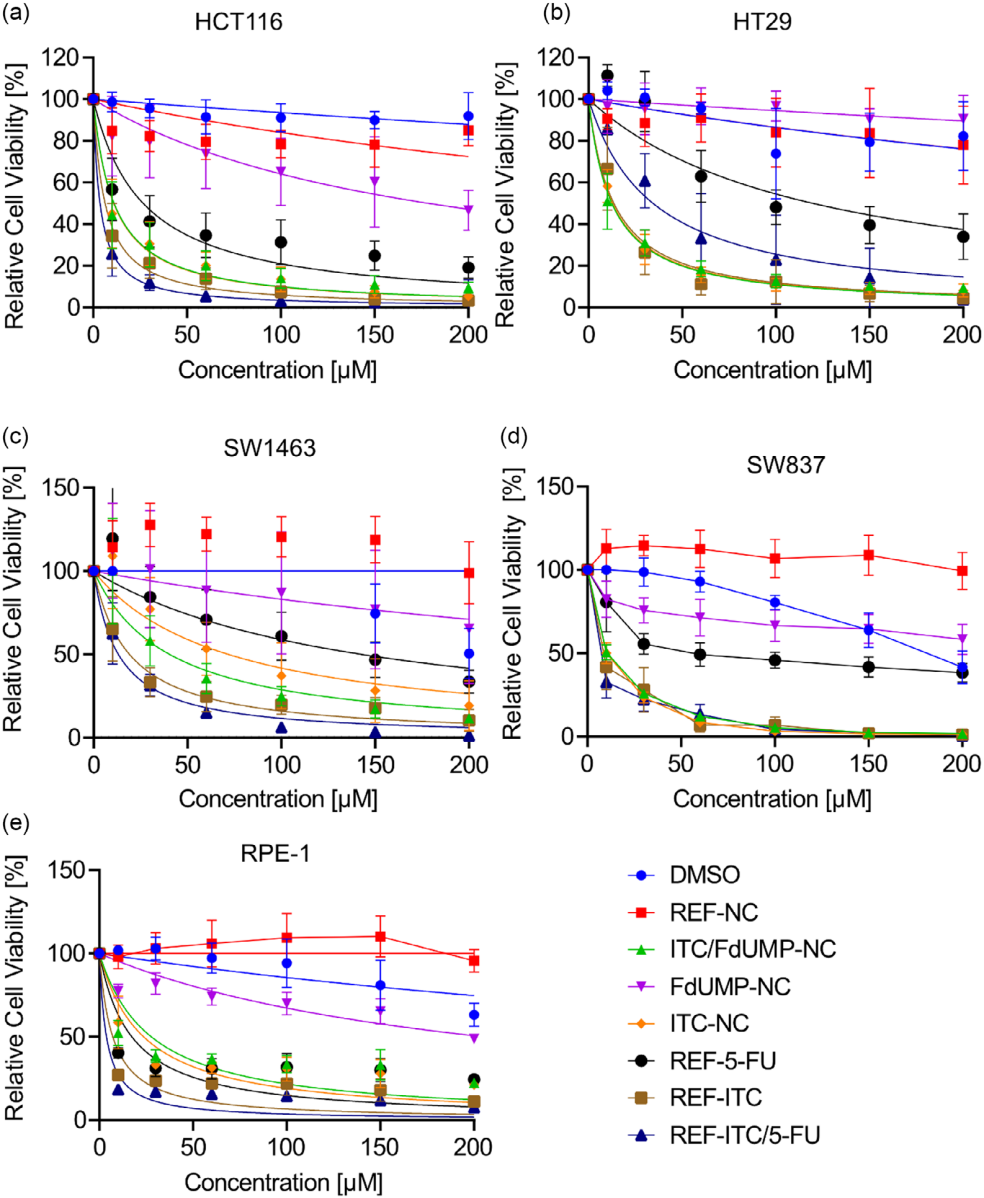
Comparative efficacy of core@shell nanocarriers and free drugs in human colon (HCT116, HT29) and rectal cancer (SW1463, SW837) as well as normal retinal epithelial (RPE‐1) cell lines: a–e) Dose‐response curves for FdUMP/5‐FU (10, 30, 60, 100, and 200 μM) and corresponding ITC concentrations (5, 8, 17, 34, 58, 87, and 116 μM) following treatment with core@shell nanocarriers (ITC‐FdUMP‐NC, ITC‐NC, FdUMP‐NC) or free drugs (REF‐ITC/5FU, REF‐ITC, REF‐5FU). Cell viability was measured at day 5 post‐treatment. DMSO served as a control for free ITC, and drug‐free REF‐NC was used as a control for core@shell nanocarriers. Data are presented as mean ± SD from three biological replicates with two technical replicates.

We assessed the cytotoxic efficacy of these NCs using CellTiter‐Glo assays, which measure ATP‐based cell viability. Dose‐response curves were generated at day 5 post‐treatment for five human CRC cell lines: two colon cancer lines (HCT116, HT29) (**Figure** **7**a,b), two rectal cancer lines (SW1463, SW837) (Figure 7c,d), and one normal epithelial cell line (RPE‐1) (Figure 7e). Treatments included drug‐free DUT647‐labeled REF‐NC, dual‐drug‐loaded NC (ITC‐FdUMP‐NC), single‐drug NC (ITC‐NC, FdUMP‐NC), and free‐drug references (REF‐ITC, REF‐5FU). DMSO, used as a vehicle for ITC, served as a control.

Except for FdUMP‐NC in HT29 cells (Figure 7b), all formulations—whether free‐drug or NC‐encapsulated chemotherapeutics—induced a dose‐dependent reduction in cell viability, though with varying efficacy across all cell lines. The free drug REF‐5FU exhibited superior cytotoxicity compared to FdUMP‐NC in all tested cell lines (Figure 7a,c–e). REF‐ITC and ITC‐NC showed similar efficacy in all cell lines (Figure 7a,b,d), except in the rectal cancer cell line SW1463 (Figure 7c) and in the normal RPE‐1 cells (Figure 7e), where REF‐ITC increased cell death than ITC‐NC.

Notably, in HT29 cells, the dual‐drug nanocarriers (ITC‐FdUMP‐NC) displayed significantly enhanced cytotoxic effects compared to the free‐drug combination (REF‐ITC‐5FU) (Figure 7b), suggesting that nanocarrier‐mediated codelivery may improve drug synergy in this cell line. In the other cell lines, ITC‐FdUMP‐NC and REF‐ITC‐5FU demonstrated comparable efficacy. Among the NC formulations, ITC‐FdUMP‐NC consistently exhibited the highest cytotoxic potency, closely matching the effects of the free‐drug combination (Figure 7a,c,d).

These findings suggest that core@shell nanocarriers provide a viable platform for effective chemotherapeutic delivery, with ITC‐FdUMP‐NC demonstrating strong cytotoxic potential. The improved efficacy in HT29 cells highlights the possibility that controlled intracellular trafficking and release dynamics contribute to an enhanced drug action. Overall, these results support the potential of nanocarrier‐based dual‐drug delivery to improve CRC treatment efficacy.

### 2.7 Drug Loaded Core@Shell Nanocarriers Show a Delayed Cytotoxic Effect Compared to Free Drugs

To gain deeper insights into the therapeutic time window of core@shell nanocarriers in comparison to free‐drug formulations in CRC, we analyzed the cellular proliferation over time, examining how these treatments influence cancer cell growth dynamics beyond their immediate cytotoxic effects. Given our findings that nanocarriers traffic through the endosomal‐lysosomal pathway before drug release, we hypothesized that this intracellular processing would influence the timing of the therapeutic response.

**Figure 8:**
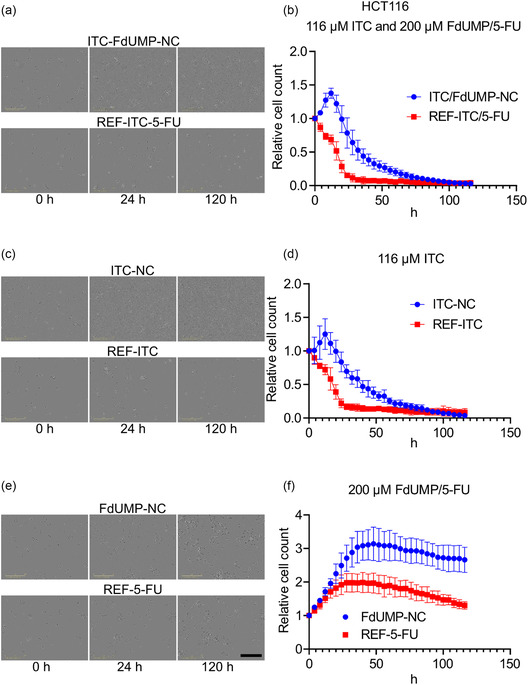
Core@shell nanocarriers show delayed and sustained cytotoxic effects: a) Representative Incucyte images of human HCT116 colon cancer cells treated with ITC‐FdUMP‐NC or free‐drug controls (REF‐ITC‐5FU, REF‐5FU) for 72 h. b) Proliferation kinetics of HCT116 cells treated with ITC‐FdUMP‐NC or REF‐ITC‐5FU, measured by live‐cell counting at 2 h intervals until 72 h using an Incucyte Live‐Cell Analysis System. c) Representative Incucyte images of HCT116 cells treated with ITC‐NC or REF‐ITC for 72 h. d) Proliferation kinetics of HCT116 cells treated with ITC‐NC or REF‐ITC, analyzed by live‐cell counting at 2 h intervals using a Incucyte Live‐Cell Analysis System. e) Representative Incucyte images of HCT116 cells treated with FdUMP‐NC or REF‐5FU for 72 h. f) Proliferation kinetics of HCT116 cells treated with FdUMP‐NC or REF‐5FU, measured by live‐cell counting at 2 h intervals until 72 h using an Incucyte Live‐Cell Analysis System. Scale bar in black color = 400 μm in all the Incucyte images. (b,d,f) Proliferation curves were generated by normalizing the cell count at each time point to the time 0 value. Data are presented as mean ± SD from three biological replicates with two technical replicates.

Using an IncucyteLive‐Cell Analysis system, we monitored cell proliferation and quantified cell counts up to 120 h to generate proliferation curves comparing the effects of NCs and free‐drug treatments in HCT116 cells (**Figure** **8**a–d).

Proliferation curves comparing ITC‐FdUMP‐NC and free ITC/5‐FU treatments revealed markedly different cytotoxicity profiles. Cells exposed to free drugs showed an immediate decline in cell number, with complete cell killing observed within ≈30 h, consistent with rapid cellular uptake and nuclear localization of the drugs. In contrast, cells treated with ITC‐FdUMP‐loaded nanocarriers initially continued to proliferate before a delayed onset of cytotoxicity emerged between 15 and 24 h after treatment. Complete cell death occurred only after ≈80 h, indicating a substantially extended therapeutic window relative to free drugs (Figure 8a,b). A similar pattern was observed for ITC‐NC compared to free ITC, with ITC‐NC‐treated cells displaying a slower but more sustained cytotoxic response (Figure 8c,d). Notably, this delayed but prolonged cytotoxic effect correlates with the slow cellular uptake and lysosome‐mediated disassembly of the nanocarriers, supporting a mechanism of sustained intracellular drug release (Figure 4,6).

**Figure 9:**
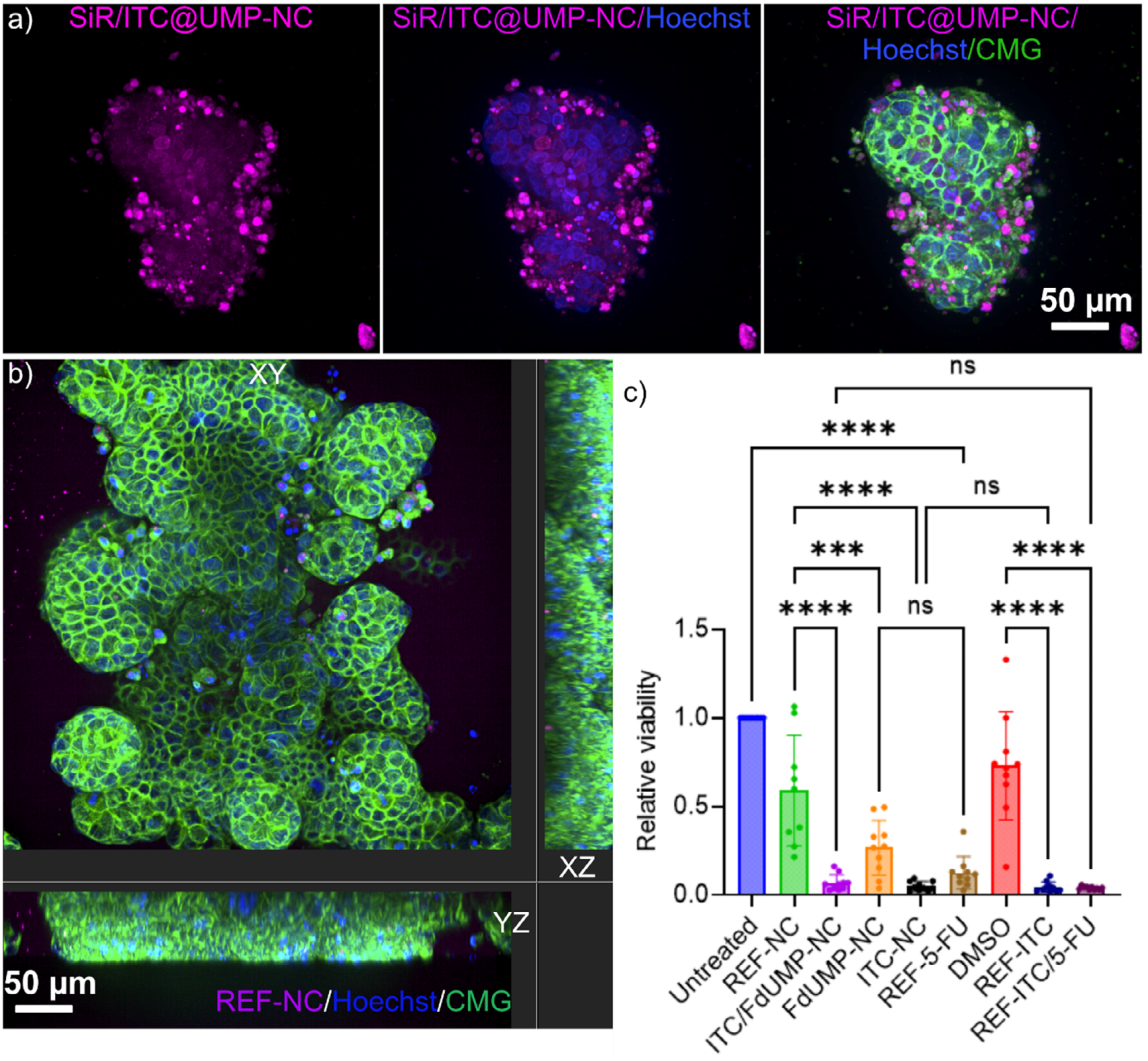
Core@shell nanocarriers exhibit equal cytotoxicity effects as free drugs in PDOs from tissue of a rectum cancer patient: a) Representative 2D spinning disk images of a PDO treated with NC for 24 h and then imaged. Nuclei are stained with 5‐SiR‐Hoechst dye (magenta), delivered via 88.05 μg mL^−1^ of SiR.0ITC‐UMP‐NC, and Hoechst (blue). The cell borders of the PDO are stained with CellMask Green (CMG). b) Representative 2D spinning disk confocal images in XY, YZ, and YZ planes of PDOs treated with 60.53 μg mL^−1^ DUT647‐labeled REF‐NC (magenta) for 24 h. Cell membranes of PDOs were stained with CMG (green) and nuclei with Hoechst (blue). c) CellTiter‐Glo viability assays of PDOs treated with controls, NCs, and free drugs (single and combination) after 5 days of treatment. Relative viability was normalized to untreated conditions. Data in b represents mean ± SD from three biological replicates; ^***^ *p* < 0.001; ^****^ *p* < 0.0001, ns = nonsignificant; one‐way ANOVA with Tukey's multiple comparisons test.

In contrast, for FdUMP‐NC and 5‐FU free drug treatments, the cellular proliferation kinetics follow a different trajectory. Rather than inducing immediate cell death, both treatments resulted in cell‐cycle arrest, consistent with the cytostatic mechanism of FdUMP.^[^ ^30^ ^]^ Similar to the ITC‐NC condition, the cytostatic effect of FdUMP‐NC is delayed compared to the free drug, further emphasizing the impact of core@shell nanocarrier uptake kinetics on drug action. While free FdUMP exerts its arresting effect more immediately upon treatment, FdUMP‐NC requires additional time to be internalized and processed within the cells before exerting its full cytostatic effect (Figure 8e,f).

Taken together, core@shell nanocarriers induce a delayed but sustained cytotoxic and cytostatic effect in CRC cells compared to free‐drug formulations. Nanocarrier‐treated cells showed a gradual decrease in cell count starting around 24 h, while free drugs caused an immediate reduction. The delayed effects of the nanocarriers are due to their slow internalization by macropinocytosis and endolysosomal processing, influencing the timing of drug release.

### 2.8 Core@Shell Nanocarriers Facilitate Efficient Drug Delivery and Cytotoxicity in Rectal Cancer Patient‐Derived Organoids

To further assess the therapeutic potential of core@shell nanocarriers, we evaluated their efficacy in a more advanced in vitro model using patient‐derived organoids (PDOs) established from rectal cancer tissue. This 3D model better recapitulates the genetic characteristics of the patient's tumor and provides a physiologically relevant platform for predicting therapy responses.^[^ ^31^ ^]^

To assess nanocarier internalization and drug release in tumor cells within the 3D PDO structure, we performed confocal microscopy using DUT647‐labeled REF‐NC and SiR/ITC‐UMP‐NC. **Figure** **9**a shows nuclear 5‐SiR‐Hoechst dye localization in a subset of cells treated with SiR/ITC‐UMP‐NC, indicating successful nanocarrier uptake and subsequent release of the dye, which reaches the nucleus. In contrast, Figure 9b confirms the efficient internalization of DUT647‐labeled REF‐NC, as indicated by the fluorescence signal within the tumor cells. These findings demonstrate that tumor cells in PDOs can take up core@shell nanocarriers and facilitate the intracellular release of their cargo to finally reach the nucleus, supporting their potential for targeted drug delivery.

After showing efficient internalization and efficient drug release, we next analyzed the therapeutic efficacy of the nanocarriers by comparing their effects on rectal cancer PDO cell viability to REF‐NC, DMSO, and free drug treatments. All tested core@shell nanocarriers significantly reduced the PDO viability compared to REF‐NC after 5 days of treatment, indicating their enhanced cytotoxic effects. Furthermore, the three core@shell nanocarriers, ITC‐NC, FdUMP‐NC, and ITC‐FdUMP‐NC, demonstrated a comparable reduction in cell viability to their respective free drug counterparts (Figure 9c). This suggests that the encapsulated drugs retained their cytotoxic potential and the nanocarriers are as effective as free drugs in inducing cell death in 3D PDO cultures.

Taken together, our data highlight the potential of core@shell nanocarriers as an effective drug‐delivery platform of drug cocktails in 3D‐tumor models, offering a promising strategy for improving chemotherapeutic outcomes.

## 3 Discussion

In this study, we demonstrate for the first time that high‐load drug‐cocktail core@shell nanocarriers (NCs) are internalized predominantly via macropinocytosis, exhibiting notably slow uptake kinetics but strong intracellular accumulation after 24 h of exposure. Despite the overall slow internalization, the internalized NCs traffic rapidly to lysosomes, with ≈75% of the NCs colocalizing with the lysosomal membrane protein LAMP1 within just 6 h. This observation reveals an uncoupling between endocytic uptake and lysosomal delivery. Using our adaptable nanocarrier‐synthesis platform, we incorporated functional probes such as MR, which confirms lysosomal trafficking and degradation. Notably, consistent with the rapid lysosomal delivery suggested by LAMP1 colocalization, a MR signal was detectable as early as 2 h after exposure. In a separate approach, encapsulating the chromatin‐binding dye 5‐SiR‐Hoechst^[^ ^27^ ^]^ enabled us to directly visualize, for the first time, the nuclear delivery of the nanocarrier cargo. Strikingly, we identified that this nuclear translocation is strongly pH‐dependent, with efficient delivery occurring only under acidic conditions, underscoring the importance of endolysosomal maturation in enabling endosomal escape and nuclear delivery. Importantly, this pH‐dependent nuclear delivery is highly relevant for the action of our encapsulated chemotherapeutics, which require both endolysosomal escape and translocation to the nucleus to exert cytotoxicity. Additionally, aiming for a higher drug payload with lipophilic and hydrophilic drugs, we achieved 57%, representing not only a significant improvement over our previous version,^[^ ^3^ ^]^ but also one of the highest payloads reported in multidrug nanocarriers. Most compellingly, we demonstrate that these ITC‐FdUMP‐based nanocarriers, in contrast to free drugs, induce a delayed but substantially prolonged cytotoxic response. Importantly, the delayed onset of cytotoxic effects at 24 h correlates with the peak in nanocarrier uptake, while the extended window of cytotoxicity aligns with the subsequent nuclear delivery of the released cargo. Thus, our findings uniquely link the mechanistic details of nanocarrier uptake and intracellular trafficking to their therapeutic outcome, providing important new insights.

By performing both confocal microscopy and EM, we observed that the core@shell nanocarriers exhibit notably slow uptake kinetics with peak uptake at 24 h. This was particularly evident when compared to transferrin, a well‐characterized ligand internalized rapidly via clathrin‐mediated endocytosis—a receptor‐dependent pathway that typically occurs within seconds.^[^ ^14^ ^]^ In contrast, our findings suggest that uptake of the nanocarriers occurs predominantly through macropinocytosis, a relatively slow, nonspecific endocytic process involving the formation of large membrane ruffles that engulf extracellular fluid into vesicles ranging from 0.2 to 5 μm.^[^ ^32^ ^]^ Given that our nanocarriers are ≈70 nm in diameter, their size aligns well with the capacity of macropinocytic vesicles. This interpretation is supported by previous studies showing that nanoparticles of various types—including ≈20 nm‐sized quantum dots and 70 nm‐sized polymeric or lipid‐based NPs—can be internalized via micropinocytosis.^[^ ^6^, ^33^, ^34^ ^]^ Notably, nab‐paclitaxel (a nanoparticle albumin‐bound formulation of paclitaxel with an average diameter of 130 nm) has also been shown to enter through this route.^[^ ^35^ ^]^ Although macropinocytosis is inherently slower than clathrin‐mediated uptake, it is often upregulated in tumor cells.^[^ ^36^ ^]^ Overall, nanoparticles of diverse matrix compositions and size ranges from 50 to 300 nm—carrying cargoes such as nucleic acids, proteins, or chemotherapeutics drugs—have been shown to enter cells through micropinocytosis.^[^ ^36^, ^37^, ^38^, ^39^, ^40^, ^41^ ^]^ It is highly possible that the dominant uptake pathway for nanocarriers varies across different cell types, with tumor cells predominantly using micropinocytosis due to their metabolic demands and altered membrane dynamics.^[^ ^42^ ^]^ Although caveolin‐1 knockdown also significantly reduced intracellular nanocarrier accumulation, we observed no colocalization of nanocarrier with caveolin‐1 protein. Furthermore, the lack of a specific inhibitor for inhibiting caveolae‐dependent endocytosis limits further validation studies.

Our colocalization analyses provide important mechanistic insights into the intracellular fate of the nanocarriers. The transient presence in early endosomes, together with the rapid appearance of strong LAMP1 colocalization within just 6 h, suggests a trafficking route that bypasses prolonged early endosomal retention—potentially minimizing premature cargo leakage. The sustained accumulation in lysosomes further supports efficient lysosomal targeting. Interestingly, we also observed an enlargement of RAB7A‐positive vesicles in treated cells, indicating that the core@shell nanocarriers not only traffic through late endosomes but may actively induce endosomal remodeling. This structural alteration of the endosomal compartments may reflect a stress response to the internalized materials or could facilitate more efficient processing and maturation toward lysosomal fusion. This remodeling might also indicate that prolonged exposure to nanocarriers could increase endosomal vesicle trafficking, which in turn may reflect more efficient lysosomal delivery of the nanocarriers. This could explain our observation that lysosomal trafficking is far more efficient than the initial uptake of the nanocarriers, suggesting that the endosomal remodeling enhances the subsequent transport and processing within lysosomes. The appearance of the MR signal after just 2 h of nanocarrier exposure—further intensified following overnight incubation—confirms rapid lysosomal delivery and processing, in line with our design strategy for stimuli‐responsive release. Notably, the results of the chromatin‐binding 5‐SiR‐Hoechst dye demonstrate successful endosomal escape and effective nuclear delivery of the nanocarrier cargo, as evidenced by the delayed yet robust nuclear accumulation compared to the rapid localization observed with the free 5‐SiR‐Hoechst.

Endosomal escape remains one of the most significant bottlenecks in intracellular drug delivery.^[^ ^5^ ^]^ Our findings suggest that the core@shell nanocarriers partially overcome this barrier, achieving a balance between sufficient lysosomal processing and successful nuclear delivery of the nanocarrier cargo. The observed delay could primarily result from slow uptake kinetics, as lysosomal trafficking occurred rapidly after internalization. Importantly, the inhibition of lysosomal acidification reduced nuclear delivery, confirming the necessity of an acidic lysosomal milieu for effective therapeutic release. The acidic disintegration of the core@shell nanocarriers within endolysosomal compartments facilitates the release of ITC and FdUMP, which subsequently diffuse across the cytoplasmic and nuclear membranes in their free drug forms. Furthermore, the increased accumulation of 5‐SiR‐Hoechst dye in the Rab7a‐positive late endosomal compartments suggests that nanocarrier entrapment in these compartments upon deacidification might hinder late endosomal fusion with lysosomes, thereby interfering with the subsequent lysosomal processing of nanocarriers required for cargo release. The dependence on lysosomal acidification suggests that the nanocarrier disassembly or cargo‐release mechanism is tightly coupled to pH‐sensitive structural transitions, highlighting the importance of endosomal microenvironment sensing for controlled intracellular release. This may involve the hydrolysis of the phosphorus‐acid ester bonding in FdUMP, the dissolution of the ions ([ZrO]^2+^, [HPO_4_]^2−^ in the particle shell, as well as of the tocopherol molecules stabilizing the interlayer between particle core and particle shell of the core@shell nanocarriers. The pH‐sensitive disassembly mechanism allows for controlled drug‐cargo release in acidic environments, such as the lysosome, where the pH is typically low (≈4.5–5.5).^[^ ^43^ ^]^ These insights into intracellular trafficking and release mechanisms are crucial for optimizing nanocarrier platforms aimed at targeted, efficient therapeutic delivery, particularly for cargoes requiring nuclear access, such as gene‐editing tools or DNA‐binding drugs.

Compared to our earlier 32 wt%‐loaded formulation,^[^ ^3^ ^]^ the current 57 wt%‐loaded nanocarriers—codelivering both irinotecan and FdUMP—significantly extend intracellular drug availability and enhance cytotoxicity, validating our rationale for increasing the overall chemotherapeutic payload. This addresses a major limitation of nanoparticle‐based drug delivery, where drug payloads often fall below 10%; for example, the clinically approved formulation nab‐paclitaxel contains only ≈10% wt paclitaxel.^[^ ^44^ ^]^ In our cell assays, such as HT29 colon cancer cells, the ITC‐FdUMP‐loaded nanocarriers induce prolonged cytotoxic responses, notably outperforming free drugs. In 3D rectal tumor patient‐derived organoids (PDOs), the nanocarriers exhibit effective tumor‐cell penetration, nuclear delivery, and therapeutic impact—further supporting their potential translational relevance.

Most importantly, proliferation kinetics revealed that the slow uptake and lysosome‐dependent disassembly of the nanocarriers translate into distinct therapeutic time windows. Unlike free drugs, which triggered immediate cytotoxic or cytostatic effects, CRC cells treated with ITC‐FdUMP‐loaded core@shell nanocarriers initially continue to proliferate, followed by a delayed but pronounced reduction in cell number only after 15 h—closely matching the slow linear uptake kinetics that peak at 24 h. Importantly, the high loading capacity of two drugs of our core@shell nanocarriers enables a substantial increase of delivered drug concentration without increasing the volume of nanoparticles applied. This results in nearly complete cytotoxicity in vitro (≈80 h post‐treatment), which was not possible with previous lower‐payload core@shell formulations.^[^ ^3^ ^]^ Such a property is crucial for in vivo applications, where limitations on total nanoparticle volume can compromise therapeutic efficacy. Therefore, this is the first time a direct link has been demonstrated between intracellular trafficking behavior and therapeutic timing. The slow and sustained release—intimately coupled with cellular uptake and processing—may also provide a clinical benefit by avoiding the high peak‐plasma concentrations associated with free‐drug toxicity. By enabling gradual, sustained drug exposure, in combination with selective tumor targeting, core@shell nanocarriers have the potential to reduce systemic side effects while maintaining therapeutic efficacy.

Our study demonstrates that these core@shell nanocarriers achieve high therapeutic efficacy not only in CRC cell lines, but also in PDOs. Given that tumor heterogeneity is a major limiting factor in chemotherapy efficacy in solid tumors, the use of PDO models is particularly important. In PDOs, tumor heterogeneity is maintained, as they closely recapitulate the histological, genetic, and transcriptional features of the original tumor.^[^ ^45^ ^]^ Previous in vivo studies from our group have already shown high tumor accumulation of comparable high‐load nanocarriers in both primary tumors and metastases in two orthotopic PDAC mouse models.^[^ ^46^ ^]^ This accumulation is likely facilitated by the enhanced permeability and retention (EPR) effect resulting from the leaky vasculature of solid tumors.^[^ ^47^, ^48^ ^]^ However, many solid tumors, such as CRC, are characterized by a dense stromal matrix that restricts deep intratumoral nanoparticle transport, leading to preferential peripheral accumulation.^[^ ^49^ ^]^ It is also important to note that the dense stromal architecture is not fully recapitulated in mouse cancer models. Orthotopic tumor‐cell implantation typically results in tumors with substantially reduced stromal content compared to human cancers.^[^ ^50^ ^]^ Furthermore, although opsonization and mononuclear phagocyte system‐mediated clearance can result in substantial off‐target liver and spleen accumulation, this was not observed in the referenced PDAC mouse study when nanocarriers were administered intraperitoneally.^[^ ^46^ ^]^ Nonetheless, for the clinical translation of our core@shell nanocarriers as a potential CRC treatment strategy, it is essential to extensively validate our in vitro findings in the future. This includes confirming their anticancer efficacy, biodistribution, and potential side effects in established human CRC xenograft mouse models.^[^ ^51^ ^]^

In conclusion, by unraveling the distinct mechanisms of nanoparticle uptake, lysosomal processing, and pH‐dependent nuclear delivery, we have demonstrated a highly efficient and sustained therapeutic response for NC‐based chemotherapeutic drug delivery. The enhanced drug payload achieved in this study, coupled with the ability to precisely target the nucleus, not only prolongs intracellular drug exposure via slow trafficking and controlled release but also permits higher dosing flexibility, expanding the therapeutic window of combination regimens like ITC and FdUMP. This offers significant potential for improving the effectiveness of nanoparticle‐based therapies. This advancement addresses a major limitation of conventional nanoparticle systems—low drug loading—which typically requires high particle doses to reach therapeutic thresholds. The elevated payload allows for treatment with markedly higher drug concentrations at substantially reduced nanoparticle volumes, a critical advantage for in vivo applications where dosing is restricted due to potential off‐target toxicity and immune‐related side effects. Beyond our specific system, the mechanistic insights gained here provide a valuable framework for the broader nanoparticle research community, where cellular uptake, intracellular trafficking, and payload release remain incompletely understood. These findings will also inform the design and interpretation of future in vivo studies, guiding the development of next‐generation nanocarriers with improved bioavailability and therapeutic efficacy.

## 4 Experimental Section

4.1

4.1.1

##### Cell Culture

All cell lines were obtained from the American Type Culture Collection (ATCC, Manassas, VA, USA). HCT116 (CVCL‐0291) and HT29 (CVCL_0320) human colon cancer cells were cultured in McCoy's medium (Gibco) supplemented with 10% fetal bovine serum (FBS) and 10 μg mL^−1^ penicillin‐streptomycin (Sigma Aldrich, P4333). RPE‐1 (CVCL_4388) human immortalized retinal pigment epithelial cells were maintained in DMEM/F12 (Gibco) with 10% FBS and 0.01 mg mL^−1^ hygromycin B. SW1463 (CVCL_1724) and SW837 (CVCL_1720) human rectal tumor cells were grown in Leibovitz's medium (Gibco) supplemented with L‐glutamine, 10% FBS, and 10 μg mL^−1^ penicillin‐streptomycin. All cells were cultured at 37 °C in a humidified incubator with 5% CO_2_ and routinely checked to ensure that they were contamination‐free.

##### Rectal Cancer Patient‐Derived Organoid (PDO) Culture

Fresh tissue samples were obtained from the University Medical Center Göttingen (UMG), Germany, with ethical approval from the UMG Ethics Committee (approval no. 25/3/17). Rectal cancer PDOs were generated and cultured following protocols described by.^[^ ^51^, ^52^ ^]^ Briefly, tumor organoids were maintained in Advanced DMEM/F12 (Gibco) supplemented with 1× GlutaMAX, 10 mM HEPES, 1× penicillin/streptomycin (10 μg mL^−1^, Gibco), 10 mM nicotinamide (Sigma), 1× B27 (Gibco), 500 nM A83‐01 (Tocris), 10 μM SB202190 (MedChemExpress), 1.25 mM N‐acetylcysteine (Sigma), 20% R‐spondin conditioned medium (produced in‐house), 10% Noggin conditioned medium (in‐house), 1× N2 supplement (Gibco), 100 μg mL^−1^ Primocin (InvivoGen), and 50 ng mL^−1^ recombinant human EGF (Gibco). Y‐27632 (10 μM; Adooq Biosciences) was added immediately following organoid isolation. For experiments, PDOs were dissociated into single cells, and 10 000 cells were embedded in 10% Matrigel (Corning) diluted in PDO tumor medium v/v and seeded into 96‐well plates. After three days of incubation to allow organoid formation and size uniformity, free drugs or core@shell nanocarriers (diluted in tumor medium) were added. PDOs were treated with NC or free drugs and the cell viability was analyzed after five days of treatment using the CellTiter‐Glo assay.

##### Cellular REF‐NC Uptake Assay

HCT116 cells were seeded at a density of 30 000 cells per well in 24‐well plates containing poly‐L‐lysine‐coated coverslips. After 48 h to allow for attachment and proliferation, cells were treated with DUT647‐labeled REF‐NC for either 4 or 24 h. For endocytosis inhibition, cells were pretreated for 2 h with either PITSTOP (20 μM, HY‐115604, MedChemExpress) or Dyngo 4a (20 μM, ab1206689, Abcam), followed by nanocarrier exposure for 4 or 24 h. For knockdown experiments, cells were transfected using Lipofectamine RNAiMAX transfection reagent (13778075, Thermo Fisher Scientific) with the following siRNAs: CAV1 (catalog no. 4390824, siRNA ID: s2446), CLTC (catalog no. 4390824, siRNA ID: s475), PICK1 (catalog no. 4392420, siRNA ID: s18129), and ANKFY1 (catalog no. 4392420, siRNA ID: s28200). A nontargeting siRNA (Negative Control No. 1, catalog no. 4390843) was included as a control. Transfected cells were incubated for 48 h prior to a 6 h treatment with DUT647‐labeled REF‐NC. Following treatment, cells were washed three times with phosphate‐buffered saline (PBS) to remove non‐internalized nanoparticles, fixed in 4% paraformaldehyde (PFA), and stained with Hoechst 33342 (62249, Thermo Fisher Scientific) and CellMask Green PM Stain (800 nM, C37608, Thermo Fisher Scientific). Imaging was performed using a Leica SP8 confocal microscope equipped with a Plan Neofluor 63× oil‐immersion objective (NA 1.4) and 1 Airy Unit pinhole. Staining and imaging settings were kept constant across conditions to ensure comparability. Quantification of core@shell nanocarrier uptake was performed using a customized CellProfiler pipeline (Broad Institute). Briefly, cells were segmented using Cell Mask Green signal as the primary object, and DUT647 fluorescence (*λ*_ex_ = 651 nm, *λ*_em_ = 673 nm) as the secondary object. Nanoparticle uptake was assessed as the number of DUT647‐positive puncta overlapping with each cell, normalized per cell.

##### Immunofluorescence Staining

A total of 30 000 cells were seeded into 24‐well plates containing poly‐L‐lysine‐coated coverslips. After two days of incubation to allow for proper attachment and cell growth, cells were treated with REF‐NC for either 4 or 24 h. For immunofluorescence staining, cells were fixed with 4% PFA and permeabilized using 0.2% Triton X‐100. Blocking was performed in 3% bovine serum albumin (BSA) in PBS, followed by a 90‐minute incubation with primary antibodies. The following primary antibodies were used: polyclonal rabbit anti‐Rab5 (1:100, PA5‐29022, Invitrogen), Coralite Plus 488‐conjugated EEA1 monoclonal antibody (1:100, CL488‐68065, Proteintech), mouse anti‐LAMP1 monoclonal antibody (H4A3, Alexa Fluor 488‐conjugated, 1:100, MA5‐18121, Invitrogen), and rabbit anti‐ANKFY1 (1:100, HPA065849, Sigma‐Aldrich). After three PBS washes, cells were incubated with Alexa Fluor 488‐ or 546‐conjugated secondary antibodies (Molecular Probes) and stained with Hoechst 33342 for nuclei visualization. Imaging conditions, including staining and microscope settings, were kept constant across all samples. Confocal microscopy was performed using a Leica SP8 system equipped with a Plan Neofluor 63× oil‐immersion objective (NA 1.4) and a 1 Airy Unit pinhole.

##### REF‐NC Colocalization Analysis with Endosomal Markers

For the colocalization analysis, the different fluorescent channels were first binarized. The threshold for the REF‐NC channel was set to the maximum gray value occurring in the *t* = 0 h image of the series, based on the assumption that this represents the maximum background intensity. The threshold for the endosomal and lysosomal markers was determined using Otsu's algorithm,^[^ ^53^ ^]^ which was applied to the image under consideration. Colocalization was considered when a pixel exceeded the threshold in both the REF‐NC channel and the respective endosomal or lysosomal channel. This analysis was performed using Python in a Jupyter notebook environment.

##### SiRNA Transfection

HCT116 cells were transfected with Silencer Select siRNAs targeting CAV1 (s2446), CLTC (s475), PICK1 (s18129), and ANKFY1 (s28200), or with Negative Control No. 1 siRNA (cat. no. 4390843; all from Thermo Fisher Scientific) using Lipofectamine RNAiMAX (Thermo Fisher Scientific) according to the manufacturer's protocol. Briefly, cells were seeded in antibiotic‐free complete growth medium to reach 40%–60% confluency at the time of transfection. siRNA‐lipid complexes were prepared in Opti‐MEM reduced serum medium and added directly to the cells to achieve a final siRNA concentration of 10 nM. Transfected cells were incubated under standard culture conditions (37 °C, 5% CO_2_) for 48 h.

##### RNA Isolation and Real‐Time qPCR

Total cellular RNA was extracted using the RNeasy reagent (Qiagen) in accordance with the manufacturer's protocol. Reverse transcription was performed using SuperScript (Invitrogen) with 1 μg of total RNA, following the supplier's instructions. The resulting cDNA was used as a template for quantitative real‐time PCR, conducted in triplicate on a CFX Connect Real‐Time PCR Detection System (Bio‐Rad) in a 25 μl reaction volume. Gene expression levels were quantified using SYBR Green I (Invitrogen), and relative transcript levels were calculated using the 2^‐ΔΔCt^ method after normalization to the housekeeping gene RPLP0.

Primers were synthesized by Metabion and used at a final concentration of 10 μM. For amplification of PICK1, the forward primer 5′‐GTGGCGAAGATGATTCAGGAGG‐3′ and reverse primer 5′‐CCTGAACTCATGTTCTCCACCAG‐3′ were used. Caveolin 1 was amplified using forward primer 5′‐CCAAGGAGATCGACCTGGTCAA‐3′ and reverse primer 5′‐GCCGTCAAAACTGTGTGTCCCT‐3′. For CLTC1, the forward primer was 5′‐GTCTTTCTCGCTACCTGGTACG‐3′ and the reverse primer was 5′‐GGTCCTGAGTCTCAGACAAAGC‐3′. Amplification of ANKFY1 utilized the forward primer 5′‐CAGTGTCTTCTGGAGTTTGGTGC‐3′ and reverse primer 5′‐TGATGACACCGTGTTGGCTGCT‐3′. The housekeeping gene RPL0 served as the internal control, with the forward primer 5′‐GATTGGCTACCCAACTGTTG‐3′ and reverse primer 5′‐CAGGGGCAGCAGCCACAAA‐3′.

##### Generation of a HCT116 Cells Stably Expressing EGFP‐RAB7A

The plasmid pLVX‐EF1a‐EGFP‐RAB7A‐IRES‐Puromycin (Addgene #133027), a lentiviral vector that allows stable integration into the host cell genome, was obtained as an agar stab from NEB bacterial stocks. The plasmid was streaked onto agar plates, cultured in liquid media, and then plasmid DNA was isolated using a standard plasmid extraction protocol. HCT116 cells were transfected with the plasmid using the lipofectamine transfection method, after 48 h, cells were selected with puromycin (1 mg mL^−1^) for 7–10 days to isolate stably transfected clones. Puromycin‐resistant colonies were then expanded and verified for EGFP‐RAB7A expression via fluorescence microscopy.

##### EGFP‐RAB7A Live‐Cell‐Imaging to Study Core@Shell Nanocarrier Degradation

5 000 cells were seeded in an 8‐well slide IBIDI chamber, after allowing proper attachment and growth for 2 days, cells were pretreated with 57.5 μg mL^−1^ of REF‐NC overnight. Non‐internalized core@shell nanocarriers were removed by washing the cells three times with PBS. Consequently, the intracellular REF‐NC degradation over time was imaged by using a Nikon spinning disk confocal microscope with a 40 × air objective for 48 h. The number of puncta per image and mean fluorescence intensity of REF‐NC over time were calculated by analyzing the time‐lapse movies of cells (brightfield) with REF‐NC fluorescent signal in three dimensions (3D) using IMARIS. To identify REF‐NC puncta and their mean fluorescence intensity, the fluorescent signal from DUT647 (*λ*_ex_ = 651 nm, *λ*_em_ = 673 nm) was segmented using the “spots” function of Imaris, followed by exporting the data into excel.

##### Measuring 5‐SiR‐Hoechst Dye Release from Core@Shell Nanocarriers

HCT116 cells stably expressing EGFP‐RAB7A were seeded in an 8‐well Ibidi chamber. To assess the release of the 5‐SiR‐Hoechst dye from endocytosed core@shell nanocarriers, cells were treated with 73.35 μg mL^−1^ of SiR/ITC‐UMP‐NC (equivalent to 25 μM of ITC) or with an equal amount of free 5‐SiR‐Hoechst dye, and then imaged for 24 h with 3 h intervals. To study the pH‐dependent release of the 5‐SiR‐Hoechst dye from endocytosed core@shell nanocarriers, cells were treated with 73.35 μg mL^−1^ of SiR/ITC‐UMP‐NC (equivalent to 25 μM of ITC) for 6 h to allow for uptake. After the incubation period, cells were thoroughly washed with fresh media to remove uninternalized nanocarriers. To monitor the effect of endolysosomal inhibition on the efficiency of dye release, cells were treated with either DMSO (control) or bafilomycin A1 (200 nM, HY‐100558, MedChemExpress) for 18 h. Following the treatments, cells were stained with Lysotracker Red DND‐99 (200 nM, Invitrogen) to label acidic compartments or with Hoechst for nuclear staining. After staining, cells were fixed with 4% PFA and prepared for imaging.

Images were acquired using a Nikon spinning disc confocal microscope with a 40x objective. The nuclear staining intensity of the 5‐SiR‐Hoechst dye, as well as the number of nuclei positive for 5‐SiR‐Hoechst, was measured per image. Total nuclei were segmented based on Hoechst staining. Image analysis was performed using CellProfiler, and the pipeline is available upon request.

##### CellTiter‐Glo Viability Assay

Cell viability was measured by performing a CellTiter‐Glo assay (Promega, G8461). Cells were seeded in a 96‐well plate. After drug treatment for the indicated duration, 80 μL of the cell titer glow reagent (Promega) diluted to 1:5 with PBS were added to each well. The plate was incubated on a shaker for 2 min at room temperature (RT) to allow cell lysis and then incubated without shaking for 10 min at RT to allow luminescence signal stabilization. The signal was measured using a multimode plate reader CLARIOstar (BMG LABTECH, Germany).

##### Incucyte Imaging and Cell Proliferation Kinetics Analysis

The real‐time proliferation of the HCT116 cells was detected by the Incucyte SX5 Live‐Cell Analysis System (Sartorius) by imaging each well at 2 h intervals for 72 h with a 10× objective. The images were then exported and the proliferation kinetics were obtained by calculating live‐cell count at each time point, normalized to the control untreated condition. We trained a custom Cellpose model^[^ ^54^ ^]^ for cell segmentation. The dataset was generated by annotating images acquired from the Incucyte system and was split into training (80%) and testing (20%) sets. To optimize the model's performance, the images were divided into overlapping 800 × 800‐pixel patches. The model was trained for 1000 epochs using transfer learning from the pretrained “live‐cell” model (batch size = 4, learning rate = 0.2). Model checkpoints were saved every 100 epochs, with the final model selection based on performance on the test set. Using the optimized algorithm, we obtained the cell count at each time point. To generate proliferation curves, we normalized the cell count at each time point to the initial time point (*t* = 0).

##### Electron Microscopy to Assess Nanocarrier Uptake in Cells

HCT116 cells (400 000 per dish) were seeded in 35 mm culture dishes. After 48 h, cells were treated with 230 μg mL^−1^ of REF‐NC for either 4 or 24 h. Following treatment, cells were fixed directly in the culture dishes using 4% formaldehyde and 2.5% glutaraldehyde in 0.1 M phosphate buffer (pH 7.4), followed by postfixation with 1% osmium tetroxide (OsO_4_; Science Services, Germany) in the same buffer at 4 °C. Samples were then dehydrated through an ethanol gradient, stained en bloc with 1.5% uranyl acetate and 1.5% tungstophosphoric acid (both from Merck, Germany) in 70% ethanol, and embedded in Epon resin (Serva, Germany). Ultrathin sections were cut parallel to the substrate using a Leica UC7 ultramicrotome and counterstained with UranylLess (Science Services, Germany). Imaging was performed using a LEO EM912 Omega transmission electron microscope (Zeiss, Germany), and digital micrographs were captured with a 2048 × 2048 on‐axis CCD camera (TRS).

##### Statistics

Data were analyzed using GraphPad Prism 10 built‐in tests. All data are presented as means ± SDs. Details about the significance test, the number of replicates, and the *P* values are reported in the respective Figure legends. Such as one‐way ANOVA with Tukey's multiple comparisons test.

## Supporting Information

Supporting Information is available from the Wiley Online Library or from the author.

## Conflict of Interest

The authors declare no conflict of interest.

## Supporting information

Supplementary Material

## Data Availability

The data that support the findings of this study are available from the corresponding author upon reasonable request.

## References

[1] N. Amreddy , A. Babu , R. Muralidharan , J. Panneerselvam , A. Srivastava , R. Ahmed , M. Mehta , A. Munshi , R. Ramesh , Adv. Cancer Res. 2018, 137, 115. 29405974 10.1016/bs.acr.2017.11.003 PMC6550462

[2] L. Huang , S. Zhao , F. Fang , T. Xu , M. Lan , J. Zhang , Biomaterials 2021, 268, 120557. 33260095 10.1016/j.biomaterials.2020.120557

[3] S. Notter , D. Choezom , T. Griebel , F. Ramos‐Gomes , W. Möbius , T. De Oliveira , L.‐C. Conradi , F. Alves , C. Feldmann , Small Sci. 2024, 4, 2400196. 40213458 10.1002/smsc.202400196 PMC11935085

[4] E. Van Cutsem , A. Cervantes , R. Adam , A. Sobrero , J. H. Van Krieken , D. Aderka , E. Aranda Aguilar, A. Bardelli, A. Benson, G. Bodoky, F. Ciardiello, A. D’Hoore, E. Diaz‐Rubio, J.‐Y. Douillard, M. Ducreux, A. Falcone, A. Grothey, T. Gruenberger, K. Haustermans, V. Heinemann, P. Hoff, C.‐H. Köhne, R. Labianca, P. Laurent‐Puig, B. Ma, T. Maughan, K. Muro, N. Normanno, P. Österlund, W. J. G. Oyen Ann. Oncol. Off. J. Eur. Soc. Med. Oncol. 2016, 27, 1386. 10.1093/annonc/mdw235 27380959

[5] J. J. Rennick , A. P. R. Johnston , R. G. Parton , Nat. Nanotechnol. 2021, 16, 266. 33712737 10.1038/s41565-021-00858-8

[6] G. Sahay , D. Y. Alakhova , A. V. Kabanov , J. Control. Release Off. J. Control. Release Soc. 2010, 145, 182. 10.1016/j.jconrel.2010.01.036 PMC2902597 20226220

[7] H. Appelqvist , P. Wäster , K. Kågedal , K. Öllinger , J. Mol. Cell Biol. 2013, 5, 214. 23918283 10.1093/jmcb/mjt022

[8] G. J. Dahe , R. S. Teotia , S. S. Kadam , J. R. Bellare , Biomaterials 2011, 32, 352. 20888631 10.1016/j.biomaterials.2010.09.005

[9] B. L. Neumeier , M. Khorenko , F. Alves , O. Goldmann , J. Napp , U. Schepers , H. M. Reichardt , C. Feldmann , ChemNanoMat. 2019, 5, 24.

[10] A. A. Thorat , S. V. Dalvi , Chem. Eng. J. 2012, 181–182, 1.

[11] R. Jog , D. J. Burgesss , J. Pharm. Sci. 2017, 106, 39. 27816266 10.1016/j.xphs.2016.09.014

[12] C. Harding , J. Heuser , P. Stahl , J. Cell Biol. 1983, 97, 329. 6309857 10.1083/jcb.97.2.329 PMC2112509

[13] A. Dautry‐Varsat , A. Ciechanover , H. F. Lodish , Proc. Natl. Acad. Sci. 1983, 80, 2258. 6300903 10.1073/pnas.80.8.2258 PMC393798

[14] M. Lakadamyali , M. J. Rust , X. Zhuang , Cell 2006, 124, 997. 16530046 10.1016/j.cell.2005.12.038 PMC2660893

[15] A. McCluskey , J. A. Daniel , G. Hadzic , N. Chau , E. L. Clayton , A. Mariana , A. Whiting , N. N. Gorgani , J. Lloyd , A. Quan , L. Moshkanbaryans , S. Krishnan , S. Perera , M. Chircop , L. von Kleist , A. B. McGeachie , M. T. Howes , R. G. Parton , M. Campbell , J. A. Sakoff , X. Wang , J.‐Y. Sun , M. J. Robertson , F. M. Deane , T. H. Nguyen , F. A. Meunier , M. A. Cousin , P. J. Robinson , Traffic Cph Den. 2013, 14, 1272. 10.1111/tra.12119 PMC4138991 24025110

[16] L. von Kleist , W. Stahlschmidt , H. Bulut , K. Gromova , D. Puchkov , M. J. Robertson , K. A. MacGregor , N. Tomilin , A. Pechstein , N. Chau , M. Chircop , J. Sakoff , J. P. von Kries , W. Saenger , H.‐G. Kräusslich , O. Shupliakov , P. J. Robinson , A. McCluskey , V. Haucke , Cell 2011, 146, 471. 21816279 10.1016/j.cell.2011.06.025

[17] K. G. Rothberg , J. E. Heuser , W. C. Donzell , Y. S. Ying , J. R. Glenney , R. G. Anderson , Cell 1992, 68, 673. 1739974 10.1016/0092-8674(92)90143-z

[18] C. Schnatwinkel , S. Christoforidis , M. R. Lindsay , S. Uttenweiler‐Joseph , M. Wilm , R. G. Parton , M. Zerial , PLoS Biol. 2004, 2, E261. 15328530 10.1371/journal.pbio.0020261 PMC514490

[19] L. Hinrichsen , J. Harborth , L. Andrees , K. Weber , E. J. Ungewickell , J. Biol. Chem. 2003, 278, 45160. 12960147 10.1074/jbc.M307290200

[20] E. Boucrot , A. P. A. Ferreira , L. Almeida‐Souza , S. Debard , Y. Vallis , G. Howard , L. Bertot , N. Sauvonnet , H. T. McMahon , Nature. 2015, 517, 460. 25517094 10.1038/nature14067

[21] M. C. Kerr , R. D. Teasdale , Traffic Cph. Den. 2009, 10, 364. 10.1111/j.1600-0854.2009.00878.x 19192253

[22] A. Simonsen , R. Lippé , S. Christoforidis , J. M. Gaullier , A. Brech , J. Callaghan , B. H. Toh , C. Murphy , M. Zerial , H. Stenmark , Nature 1998, 394, 494. 9697774 10.1038/28879

[23] G. Griffiths , B. Hoflack , K. Simons , I. Mellman , S. Kornfeld , Cell 1988, 52, 329. 2964276 10.1016/s0092-8674(88)80026-6

[24] P. A. Vanlandingham , B. P. Ceresa , J. Biol. Chem. 2009, 284, 12110. 19265192 10.1074/jbc.M809277200 PMC2673280

[25] Ala‐Pro‐Cresyl Violet, a Synthetic Fluorogenic Substrate for the Analysis of Kinetic Parameters of Dipeptidyl Peptidase IV (CD26) in Individual Living Rat Hepatocytes. 10.1006/abio.1997.2312 9324943

[26] D. C. Barral , L. Staiano , C. Guimas Almeida , D. F. Cutler , E. R. Eden , C. E. Futter , A. Galione , A. R. A. Marques , D. L. Medina , G. Napolitano , C. Settembre , O. V. Vieira , J. M. F. G. Aerts , P. Atakpa‐Adaji , G. Bruno , A. Capuozzo , E. De Leonibus , C. Di Malta , C. Escrevente , A. Esposito , P. Grumati , M. J. Hall , R. O. Teodoro , S. S. Lopes , J. P. Luzio , J. Monfregola , S. Montefusco , F. M. Platt , R. Polishchuck , M. De Risi , et al., Traffic Cph. Den. 2022, 23, 238. 10.1111/tra.12839 PMC9323414 35343629

[27] J. Bucevičius , J. Keller‐Findeisen , T. Gilat , S. W. Hell , G. Lukinavičius , Chem. Sci. 2019, 10, 1962. 30881625 10.1039/c8sc05082a PMC6385482

[28] T. Yoshimori , A. Yamamoto , Y. Moriyama , M. Futai , Y. Tashiro , J. Biol. Chem. 1991, 266, 17707. 1832676

[29] K. W. Beyenbach , H. Wieczorek , J. Exp. Biol. 2006, 209, 577. 16449553 10.1242/jeb.02014

[30] S. K. Jain , A. Jain , Expert Opin. Drug Deliv. 2008, 5, 483. 18491977 10.1517/17425247.5.5.483

[31] G. Vlachogiannis , S. Hedayat , A. Vatsiou , Y. Jamin , J. Fernández‐Mateos , K. Khan , A. Lampis , K. Eason , I. Huntingford , R. Burke , M. Rata , D.‐M. Koh , N. Tunariu , D. Collins , S. Hulkki‐Wilson , C. Ragulan , I. Spiteri , S. Y. Moorcraft , I. Chau , S. Rao , D. Watkins , N. Fotiadis , M. Bali , M. Darvish‐Damavandi , H. Lote , Z. Eltahir , E. C. Smyth , R. Begum , P. A. Clarke , J. C. Hahne , et al., Science 2018, 359, 920. 29472484 10.1126/science.aao2774 PMC6112415

[32] J. A. Swanson , C. Watts , Trends Cell Biol. 1995, 5, 424. 14732047 10.1016/s0962-8924(00)89101-1

[33] L. W. Zhang , N. A. Monteiro‐Riviere , Toxicol. Sci. Off. J. Soc. Toxicol. 2009, 110, 138. 10.1093/toxsci/kfp087 19414515

[34] M. Hussain , M. Shchepinov , M. Sohail , I. F. Benter , A. J. Hollins , E. M. Southern , S. Akhtar , J. Control. Release Off. J. Control. Release Soc. 2004, 99, 139. 10.1016/j.jconrel.2004.06.009 15342187

[35] J. Cullis , D. Siolas , A. Avanzi , S. Barui , A. Maitra , D. Bar‐Sagi , Cancer Immunol. Res. 2017, 5, 182. 28108630 10.1158/2326-6066.CIR-16-0125 PMC5570452

[36] C. Commisso , S. M. Davidson , R. G. Soydaner‐Azeloglu , S. J. Parker , J. J. Kamphorst , S. Hackett , E. Grabocka , M. Nofal , J. A. Drebin , C. B. Thompson , J. D. Rabinowitz , C. M. Metallo , M. G. Vander Heiden , D. Bar‐Sagi , Nature 2013, 497, 633. 23665962 10.1038/nature12138 PMC3810415

[37] J. L. Huang , G. Jiang , Q. X. Song , X. Gu , M. Hu , X. L. Wang , H.‐H. Song , L.‐P. Chen , Y.‐Y. Lin , D. Jiang , J. Chen , J.‐F. Feng , Y.‐M. Qiu , J.‐Y. Jiang , X.‐G. Jiang , H.‐Z. Chen , X.‐L. Gao , Nat. Commun. 2017, 8, 15144. 28489075 10.1038/ncomms15144 PMC5436231

[38] M. Liu , Q. Li , L. Liang , J. Li , K. Wang , J. Li , M. Lv , N. Chen , H. Song , J. Lee , J. Shi , L. Wang , R. Lal , C. Fan , Nat. Commun. 2017, 8, 15646. 28561031 10.1038/ncomms15646 PMC5460036

[39] K. T. Love , K. P. Mahon , C. G. Levins , K. A. Whitehead , W. Querbes , J. R. Dorkin , J. Qin , W. Cantley , L. L. Qin , T. Racie , M. Frank‐Kamenetsky , K. N. Yip , R. Alvarez , D. W. Y. Sah , A. de Fougerolles , K. Fitzgerald , V. Koteliansky , A. Akinc , R. Langer , D. G. Anderson , Proc. Natl. Acad. Sci. 2010, 107, 1864. 20080679 10.1073/pnas.0910603106 PMC2804742

[40] H. Meng , S. Yang , Z. Li , T. Xia , J. Chen , Z. Ji , H. Zhang , X. Wang , S. Lin , C. Huang , Z. H. Zhou , J. I. Zink , A. E. Nel , ACS Nano 2011, 5, 4434. 21563770 10.1021/nn103344k PMC3125420

[41] C. Tapeinos , G. Torrieri , S. Wang , J. P. Martins , H. A. Santos , J. Control. Release 2023, 362, 225. 37625597 10.1016/j.jconrel.2023.08.045

[42] Y. X. Li , H. B. Pang , J. Control. Release Off. J. Control. Release Soc. 2021, 329, 1222. 10.1016/j.jconrel.2020.10.049 PMC7905157 33622520

[43] P. Saftig , J. Klumperman , Nat. Rev. Mol. Cell Biol. 2009, 10, 623. 19672277 10.1038/nrm2745

[44] N. P. Desai , V. Trieu , L. Y. Hwang , R. Wu , P. Soon‐Shiong , W. J. Gradishar , Anticancer Drugs. 2008, 19, 899. 18766004 10.1097/CAD.0b013e32830f9046

[45] D. Wang , R. Villenave , N. Stokar‐Regenscheit , H. Clevers , Nat. Rev. Drug Discov. 2025, 1. 41225057 10.1038/s41573-025-01317-y

[46] M. Ischyropoulou , K. Sabljo , L. Schneider , C. M. Niemeyer , J. Napp , C. Feldmann , F. Alves , Adv. Mater. 2023, 35, 2305151. 10.1002/adma.202305151 37587542

[47] Y. Matsumura , H. Maeda , Cancer Res. 1986, 46, 6387. 2946403

[48] H. Kobayashi , R. Watanabe , P. L. Choyke , Theranostics 2013, 4, 81. 24396516 10.7150/thno.7193 PMC3881228

[49] L. Miao , C. M. Lin , L. Huang , J. Control. Release 2015, 219, 192. 26277065 10.1016/j.jconrel.2015.08.017 PMC4656082

[50] M. V. Guerin , V. Finisguerra , B. J. Van den Eynde , N. Bercovici , A. Trautmann , eLife 2020, 9, e50740. 31990272 10.7554/eLife.50740 PMC6986875

[51] T. De Oliveira , T. Goldhardt , M. Edelmann , T. Rogge , K. Rauch , N. D. Kyuchukov , K. Menck , A. Bleckmann , J. Kalucka , S. Khan , J. Gaedcke , M. Haubrock , T. Beissbarth , H. Bohnenberger , M. Planque , S.‐M. Fendt , L. Ackermann , M. Ghadimi , L.‐C. Conradi , Cancers 2021, 13, 1011. 33671096 10.3390/cancers13051011 PMC7957803

[52] T. Sato , D. E. Stange , M. Ferrante , R. G. J. Vries , H. Clevers , Gastroenterology 2011, 141, 1762. 21889923 10.1053/j.gastro.2011.07.050

[53] N. Otsu , IEEE Trans. Syst. Man Cybern. 1979, 9, 62. 10.1109/tsmc.1979.4310068 10242122

[54] C. Stringer , T. Wang , M. Michaelos , M. Pachitariu , Nat Methods 2021, 18, 100. 33318659 10.1038/s41592-020-01018-x

