# Supplementary material for: Macropinocytic Uptake and pH‐Responsive Endolysosomal Processing Drive Sustained Chemotherapeutic Efficacy of High‐Load Core@Shell Nanocarriers in Colorectal Cancer
Source: Small Sci. 2025 Dec 15;6(1):e202500470. doi: 10.1002/smsc.202500470 (PMC12794672; doi:10.1002/smsc.202500470)
Supplement: Supplementary file 1 — Supplementary Material [file SMSC-6-e202500470-s001.zip › smsc70197-sup-0001-SuppData-S1.pdf]

Supplementary information

Macropinocytic uptake and pH-responsive endolysosomal processing drive sustained chemotherapeutic efficacy of high-load core@shell nanocarriers in colorectal cancer

Dolma Choezom[1,2,3], Silke Notter[4], Titus Griebel[1,3], Nathalia Ferreira[3], Johann Gruetz[2,3], Ajinkya Kulkarni[3], Matthias Schröter[3], Gražvydas Lukinavičius[5], Wiebke Möbius[6], Lena-Christin Conradi[2], Claus Feldmann[4*], Frauke Alves[1,3,7*]

[1] Translational Molecular Imaging, Clinic for Haematology and Medical Oncology, University Medical Center Goettingen (UMG), Goettingen, Germany

[2] Department of General, Visceral and Pediatric Surgery, University Medical Center Goettingen (UMG), Goettingen, Germany

[3] Translational Molecular Imaging, Max-Planck-Institute for Multidisciplinary Sciences (MPI-NAT), City Campus, Goettingen, Germany

[4] Institute of Inorganic Chemistry, Karlsruhe Institute of Technology (KIT), Karlsruhe, Germany

[5] Chromatin Labelling and Imaging Group, Max-Planck-Institute for Multidisciplinary Sciences (MPI-NAT), Goettingen, Germany

[6] Department of Neurogenetics, Max-Planck-Institute for Multidisciplinary Sciences (MPI-NAT), Goettingen, Germany

[7] Translational Molecular Imaging, Institute for Clinical and Interventional Radiology, University Medical Center Goettingen (UMG), Goettingen, Germany.

*Corresponding authors

1. Analytical techniques for material characterization

Scanning electron microscopy (SEM)

Scanning electron microscopy (SEM) was carried out with a Zeiss Supra 40 VP microscope (Zeiss, Germany), equipped with a Schottky field emitter (2.0 nm resolution). To this concern, diluted aqueous suspensions of the ITC/TocP@ZrO(FdUMP) core@shell nanocarriers were deposited on silicon wafers and left for drying overnight. The acceleration voltage was 5 kV and the working distance was 2-3 mm. Average particle diameters were calculated by statistical evaluation of at least 100 nanoparticles (ImageJ 1.47v software).

Transmission electron microscopy (TEM)

Transmission electron microscopy (TEM) and high-angle annular dark-field scanning transmission electron microscopy (HAADF-STEM) were conducted with a FEI Osiris microscope at 200 kV (FEI, The Netherlands). TEM samples were prepared by evaporating aqueous suspensions of the ITC/TocP@ZrO(FdUMP) core@shell nanocarriers on amorphous carbon (Lacey-)film suspended on copper grids.

Energy-dispersive X-ray spectroscopy (EDXS)

High-resolution EDXS was performed to analyze the chemical composition of single ITC/TocP@ZrO(FdUMP) core@shell nanocarriers. The spectra were obtained at 200 kV electron energy with a FEI Osiris microscope that was equipped with a Bruker Quantax system (XFlash detector, Bruker, Germany). EDX spectra were quantified with the FEI software package “TEM imaging and analysis” (TIA). Using TIA, element concentrations were calculated on the basis of a refined Kramers’ law model that includes corrections for detector absorption and background subtraction. Standardless quantification, i.e. by means of theoretical sensitivity factors, without thickness correction was applied. EDX spectra were taken in the STEM mode with a probe diameter of 0.5 nm. Using a focused electron probe, EDXS area scans were performed to obtain average compositions of larger sample regions. The EDX spectra were acquired by continuously scanning the electron probe in the pre-defined region.

Dynamic light scattering (DLS)

DLS was used to determine the hydrodynamic diameter of the as-prepared ITC/TocP@ZrO(UMP) core@shell nanocarriers in aqueous suspension. Studies were

conducted at room temperature in polystyrene cuvettes applying a Nanosizer ZS (Malvern Instruments, United Kingdom).

Zeta-potential analysis

Zeta potential measurements were performed using an automatic MPT-2 titrator attached to the mentioned Nanosizer ZS (Malvern Instruments, United Kingdom). For measurements, 1 mL of the aqueous suspension containing 4.3 mg/mL of the ITC/TocP@ZrO(UMP) core@shell nanocarriers were diluted in 10 mL of demineralized water and titrated with 0.1 M HCl, 0.1 M NaOH and 0.01 M NaOH.

X-ray powder diffraction (XRD)

X-ray powder diffraction (XRD) was performed with a Stoe STADI-MP diffractometer (Stoe, Germany) operating with Ge-monochromatized Cu-Kα-radiation (λ = 1.54178 Å) and Debye-Scherrer geometry. The dried ITC/TocP@ZrO(UMP) core@shell nanocarriers were fixed between Scotch tape and acetate paper and measured between -69° and +69° of two-theta.

Fourier-transformed infrared spectroscopy (FT-IR)

Fourier-transformed infrared spectroscopy (FT-IR) was performed on a Bruker Vertex 70 FT-IR spectrometer (Bruker, Germany). All nanocarrier samples and references were pestled and diluted with KBr (3 mg of sample per 300 mg of KBr) and pressed to pellets.

Elemental analysis (C/H/N/S analysis)

Elemental analysis (C/H/N/S analysis) was performed via thermal combustion with an Elementar Vario Microcube device (Elementar, Germany) at a temperature of about 1100 °C. Values were corrected for solvent content. Starting materials were measured for calculation, resulting in 66.4wt.-% C, 6.1 wt.-% H, 9.1 wt.-% N for ITC; 57.9 wt.-% C, 8.7 wt.-% H, 0.0 wt.-% N for TocP; 23.1 wt.-% C, 5.3 wt.-% H, 7.1 wt.-% N for UMP.

Optical spectroscopy (UV-Vis spectroscopy)

UV-Vis spectroscopy was used to quantify the amount of ITC and UMP in the respective ITC/TocP@ZrO(FdUMP) core@shell nanocarriers according to the Kubelka-Munk formalism. UV-VIS spectra were recorded with an UV2700 from Shimadzu (Japan). Nanoparticle suspensions were measured in polystyrene cuvettes in an integrating sphere in diffuse transmission geometry against the corresponding pure solvent as a reference. Calibration curves were measured following the Lambert-

Beer law for ITC/TocP suspensions with concentrations of 16.05, 10.70, 4.82, 2.85, 1.61 µg/mL and an absorbance (at 360 nm) of 0.380, 0.250, 0.116, 0.066, 0.037 resulting in a calibration curve gradient of 0.02351 µg–1mL–1. The absorbance ratio of 260 nm/360 nm was determined to be 1.1. For UMP, solutions with concentrations of 16.70, 5.60, 1.85, 0.21 µg/mL and an absorbance (at 260 nm) of 0.353, 0.118, 0.042, 0.005, resulting in a calibration curve gradient of 0.0212 µg–1mL–1 were measured. ITC/TocP@ZrO(FdUMP) core@shell nanocarriers were measured with a dilution factor of 300 (ITC quantification at 360 nm: absorbance 0.168). The mathematical residual absorbance obtained through the absorbance ratio 260 nm/360 nm gave the UMP quantification (UMP quantification at 260 nm: absorbance: 0.340; mathematical residual absorbance: 0.160).

Fluorescence spectroscopy

Fluorescence was studied with a Horiba Jobin Yvon Spex Fluorolog 3 (Horiba Jobin Yvon, France) equipped with a 450 W Xe-lamp. Nanoparticle suspensions were measured in polystyrene cuvettes.

2. Synthesis of nanocarriers and references

General aspects

Irinotecan (98%, ABCR, Germany), dimethyl sulfoxide (≥ 99.5%, Sigma-Aldrich, Germany), (±)-α-tocopherol phosphate disodium salt (≥ 97%, Sigma-Aldrich), ammonium acetate (97%, VWR, Germany), zirconyl chloride octahydrate (> 99 %, Sigma-Aldrich), uridine-5´-monophosphate disodium salt (99 %, Thermo Fisher Scientific, Germany), 5-fluoro-2´-deoxyuridine 5´-monophosphate sodium salt (~85 %, Sigma-Aldrich), trisodium citrate (99%, Carl-Roth, Germany), fluorescence red (Kremer Pigmente, Germany), Magic Red® MR-FR2 (Immunochemistry technologies, USA), 5-SiR-Hoechst (44) and DYTM-647P1-aadUTP (Dyomics, Germany) were used as purchased.

ITC/TocP@ZrO(FdUMP) and ITC/TocP@ZrO(UMP) nanocarriers

First, the ITC core was prepared using a solvent-antisolvent approach by adding 2.2 mg (3.75 mmol, 1.00 eq.) of ITC in 0.15 mL of DMSO as the solvent and 2.0 mg (3.61 mmol, 0.96 eq.) of TocP in 10.0 mL of demineralised water and subsequent addition of 15.0 mg (195 mmol, 52.0 eq.) of ammonium acetate as the antisolvent. Fast injection of the solvent into the antisolvent with ultrasound (Badelin Sonopuls HD 2070, 20 kHz, 70 W; 10 s, amplitude 100%) for intense mixing in an ice bath resulted in a colourless

suspension. Thereafter, two drops of an aqueous $ZrOCl_2 \times 8H_2O$ solution (1 mg/mL) were added. Simultaneously, 3.5 mL (3.5 mg, 19.7 mmol, 5.25 eq.) an aqueous $ZrOCl_2 \times 8H_2O$ solution and 3.5 mL (3.5 mg, 10.8 mmol, 2.88 eq.) of an aqueous NaH(FdUMP) solution for ITC/TocP@ZrO(FdUMP) nanocontainers or 3.5 mL (3.5 mg, 10.8 mmol, 2.88 eq.) of an aqueous Na2(UMP) solution for ITC/TocP@ZrO(UMP) nanocontainers were added. After centrifugation (25,000 rpm, 55,201×g, 15 min), the solid was dried or resuspended in trisodium citrate solution (1.0 mg/mL).

ZrO(FdUMP) and ZrO(UMP) nanocontainers (monodrug references)

100 µL of an aqueous solution of $ZrOCl_2 \times 8H_2O$ (5.80 mg, 18.0 mmol, 1.00 eq.) were injected into 10 mL of an aqueous solution of NaH(FdUMP) (7.36 mg, 22.7 mmol, 1.17 eq.) to obtain ZrO(FdUMP) nanocontainers or into 10 mL of an aqueous solution of Na_2(UMP) (7.36 mg, 22.7 mmol, 1.17 eq.) to obtain ZrO(UMP) nanocontainer. After 2 min of intense stirring, the ZrO(FdUMP) or ZrO(UMP) nanocontainer were separated by centrifugation (25,000 rpm, 55,201×g, 15 min) and purified by redispersion/centrifugation in/from H_2O. After resuspension the ZrO(FdUMP) and ZrO(UMP) nanocontainers were obtained as colloidally stable, colorless suspensions.

Fluorescence labelling with FR and DUT647

For biological studies on efficacy and cellular uptake, the nanocontainers were fluorescence labeled by addition of small amounts of the fluorescent dyes FR or DUT647. For core labelling of ITC/TocP@ZrO(UMP) and ITC/TocP@ZrO(FdUMP) nanocontainers, a solution of 75.0 µg FR (69.5×10^{-6} mmol, 18.5×10^{-6} eq.) in 0.15 mL DMSO was used instead of pure DMSO as solvent. For shell labelling of ITC/TocP@ZrO(UMP) and ITC/TocP@ZrO(FdUMP) nanocontainers, 12.2 µg DUT647 (10.0×10^{-6} mmol, 2.67×10^{-6} eq.) were added to the Na_2(UMP) or NaH(FdUMP) solution. ZrO(UMP) and ZrO(FdUMP) nanocontainers were labelled by addition of 48.8 µg DUT647 (40.0×10^{-6} mmol, 10.7×10^{-6} eq.) to the Na_2(UMP) or the NaH(FdUMP) solution. The resulting suspensions exhibited magenta-coloured (FR) or blue-colourd (DUT647) emission, which is characteristic for the respective dye.

Fluorescence labelling with MR and 5-SiR-Hoechst

First, the ITC core was prepared using a solvent-antisolvent approach by adding 2.3 mg (3.92 mmol, 1.00 eq.) of ITC in 0.15 mL MR-DMSO (MR for 10 tests in 0.15 mL DMSO) or 5-SiR-Hoechst-DMSO (19.5 µg, 2.05×10^5 mmol, 5.24×10^6 eq. in 0.15 mL DMSO) as the solvent and 3.9 mg (7.03 mmol, 1.79 eq.) of TocP in 12.0 mL of

demineralized water and subsequent addition of 30.0 mg (390 mmol, 99.4 eq.) of ammonium acetate as the antisolvent. Fast injection of the solvent into the antisolvent with ultrasound (Badelin Sonopuls HD 2070, 20 kHz, 70 W; 10 s, amplitude 100%) for intense mixing in an ice bath resulted in a colourless suspension. Thereafter, 9.0 mL (6.75 mg, 21.0 mmol, 5.35 eq.) of an aqueous $ZrOCl_2 \times 8H_2O$ solution were added dropwise. After a step of centrifugation (25,000 rpm, 55,201×g , 15 min), the solid was resuspended in 6 mL of an ammonium acetate solution (30 mg, 390 mmol, 99.4 eq) with ultrasonic irradiation (Badelin Sonopuls HD 2070, 20 kHz, 70 W; 30 s, amplitude 80%). During another ultrasonic treatment (Badelin Sonopuls HD 2070, 20 kHz, 70 W; 30 s, amplitude 50%), 2.1 mL of a solution of NaH(FdUMP) (1.0 mg, 3.07 mmol, 0.78 eq) were injected to obtain ITC/TocP@ZrO(FdUMP) nanocarriers, or 2.1 mL of a solution of Na_2(UMP) (1.0 mg, 3.07 mmol, 0.78 eq) were injected to obtain ITC/TocP@ZrO(UMP) nanocarriers after 10 s. After centrifugation (25,000 rpm, 55,201×g, 15 min), the solid was dried or resuspended in trisodium citrate solution (1.0 mg/mL).

3. Material characterization of ITC/TocP@ZrO(FdUMP) core@shell nanocarriers

In contrast to our previously reported ITC@ZrO(TocP)/ZrO(FdUMP) core@shell nanocontainers, which contained only 22 wt-% ITC and 10 wt-% FdUMP as well as an insufficient ITC : FdUMP ratio of 2.2 : 1.0 in regard of achieving optimal synergistic effects,[1] the here shown synthesis strategy for the first time allowed to obtain significantly higher drug loads of 28 wt-% ITC and 29 wt-% FdUMP as well as a an ITC : FdUMP ratio of 1 : 1.8, which is much more relevant for clinical application (see main paper: Figure 1 and Figure 2).

To enable fluorescence tracking of the ITC/TocP@ZrO(FdUMP) core@shell nanocarriers for both *in vitro* and *in vivo* applications, fluorescence labeling is possible either in the lipophilic ITC core or in the hydrophilic ZrO(FdUMP) shell. This is accomplished by adding a small amount of a suitable fluorescent dye ($0.1\text{-}1.9 \times 10^{-3}$ mol-%) during synthesis to the corresponding ITC or FdUMP solution. In particular, we employed the lipophilic perylene-based dye Fluorescence Red (FR) for ITC, and/or the hydrophilic triphosphate-functionalized dye DY-647P1-aadUTP (DUT647) for FdUMP (Figure S1a). Both dyes exhibit strong emission. The successful integration of the dyes into the ITC/TocP@ZrO(FdUMP) core@shell nanocarriers was visually already evidenced by the color of the resulting suspensions: FR-labeled nanocarriers show a

red appearance, while DUT647-labeled nanocarriers display a bluish color (Figure S1b). The presence of the dyes was further confirmed by fluorescence spectroscopy. The FR-labeled nanocarriers exhibit excitation at 480-580 nm (λ_{max}: 575 nm) and emission within 580-700 nm (λ_{max}: 615 nm). In contrast, DUT647-labeled nanocarriers display excitation in the 580-660 nm range (λ_{max}: 647 nm) and emission at 660-750 nm (λ_{max}: 670 nm) (Figure S1c).

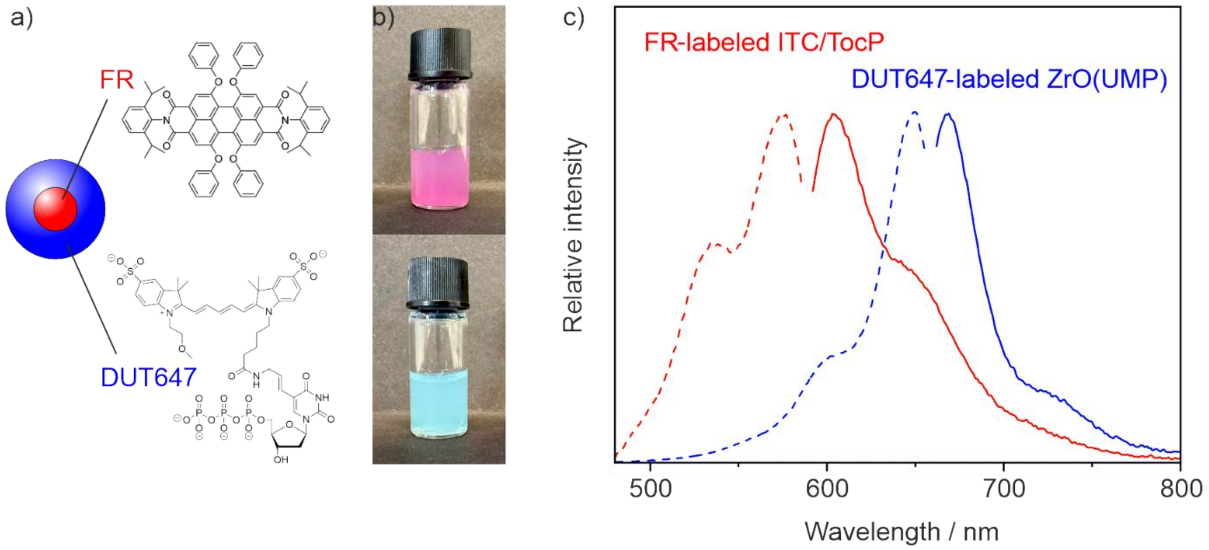

Figure S1: Fluorescence of FR-labeled (1.9×10^{-3} mol-%) and DUT647-labeled (0.1×10^{-3} mol-%) ITC/TocP@ZrO(UMP) nanocarriers: **(a)** scheme of core@shell nanocarrier structure and the localization of the respective dye; **(b)** photos of suspensions; **(c)** excitation and emission spectra with λ_{ex}: 575, λ_{em}: 615 nm for FR and λ_{ex}: 647, λ_{em}: 670 nm for DUT647.

X-ray powder diffraction (XRD) revealed the ITC@ZrO(TocP)/ZrO(FdUMP) core@shell nanocontainers to be amorphous (Figure S2). In fact, non-crystallinity is to be expected considering the large size of the drug anions and the room-temperature synthesis.

Fourier-transform (FT-IR) spectra qualitatively prove the presence of ITC, FdUMP/UMP, and TocP in the nanocarriers (Figure S3). Thus, the most characteristic vibrations can be attributed to the functional groups of the respective species, including the P=O stretching vibrations (1100, 990 cm^{-1}) originating from FdUMP/UMP and TocP, as well as the C=O stretching vibrations (1680 cm^{-1}) originating from UMP. Additionally, pyridine-related vibrations originating from ITC are observed in the region between 1620 and 1600 cm^{-1} (ν(C=N), ν(C=C)).

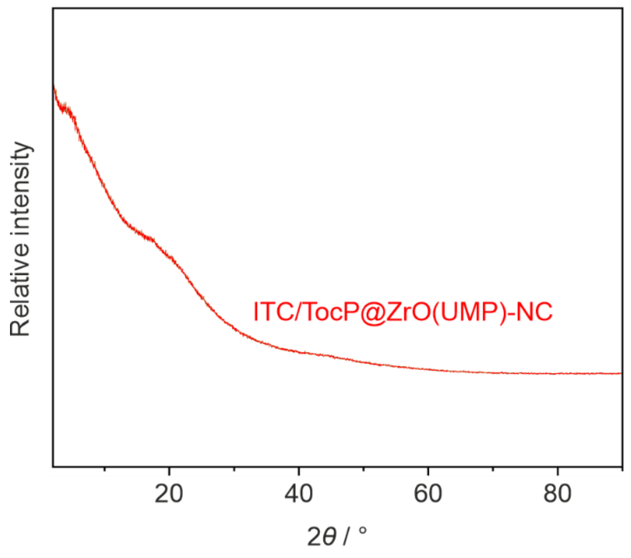

Figure S2: XRD of as-prepared ITC/TocP@ZrO(UMP) core@shell nanocarriers.

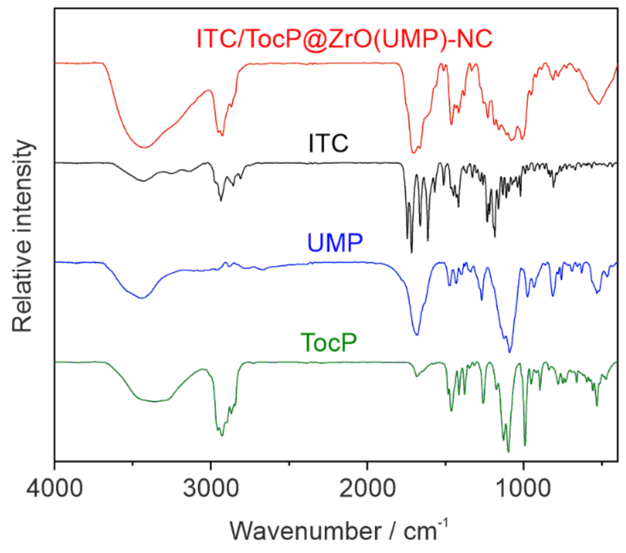

Figure S3: FT-IR spectrum of the as-prepared ITC/TocP@ZrO(UMP) core@shell nanocarriers (with spectra of pure ITC, UMP, TocP as references).

To quantify the chemical composition of the ITC/TocP@ZrO(FdUMP) core@shell nanocarriers and their drug load, total organics combustion with thermogravimetry (TG), elemental analysis (EA) and photometry were applied (*see main paper*). Due to the high costs of NaH(FdUMP) (100 mg á 4,000 €), the analytical characterization was performed with ITC/TocP@ZrO(UMP) core@shell nanocarriers and the much cheaper Na_2(UMP) (100 mg á 5 €). In difference to FdUMP, UMP does not contain any fluorine and is not cytotoxic, which, however, does not affect particle size or the overall composition of the nanocarriers.

TG shows the removal of water up to 130 °C (7 wt-%) and a total organic combustion up to 1200 °C with a total mass loss of 68 wt-% due to complete decomposition of ITC, TocP and UMP (Figure S4a). The solid residue (25 wt-%) was identified by XRD as a mixture of ZrO_2 ZrP_2O_7 and $Zr_2P_2O_9$ (Figure S4b).

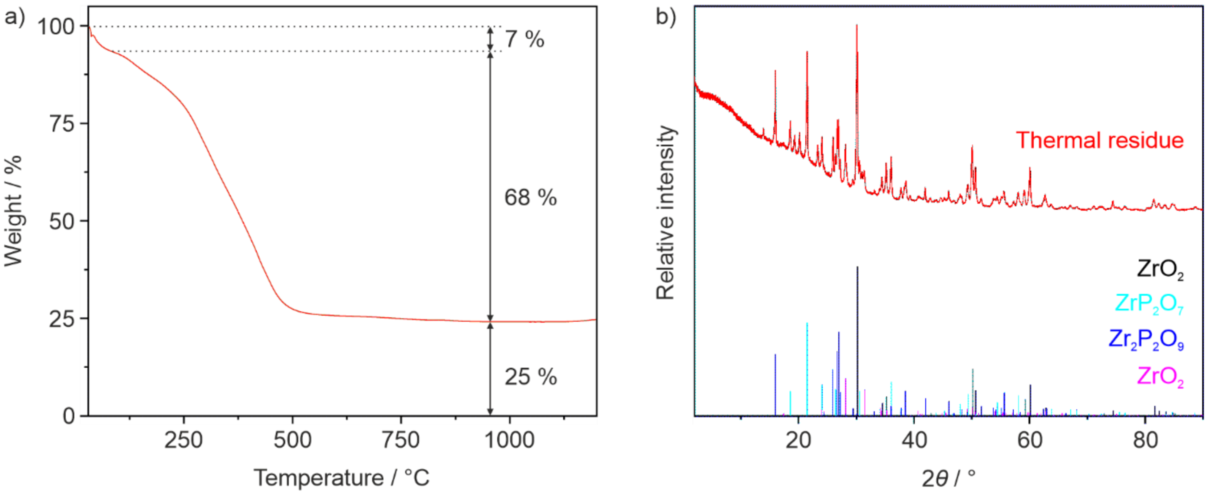

Figure S4: Total organic combustion with TG of the as-prepared ITC/TocP@ZrO(UMP) core@shell nanocarriers: **(a)** TG analysis, **(b)** XRD of the thermal remnant (references: ZrO_2/ICDD-No. 01-072-7115, ZrO_2/ICDD-No. 01-070-2491, ZrP_2O_7/ICDD-No. 00-049-1079, $Zr_2P_2O_9$/ICDD-No. 00-038-0018).

The drug load of the ITC/TocP@ZrO(UMP) nanocarriers was quantified by EA (*see main paper*) and photometry. For photometric analysis, UV-Vis spectra were recorded to employ the characteristic absorbance of ITC (360 nm) and UMP (260 nm) and to to compare with suitable references of known concentration according to the Lambert-Beer equation (Figure S5a). The reference samples containing ITC/TocP suspended in water and UMP dissolved in water demonstrate a clear linear relationship between optical absorbance and concentration (Figure S5b). Using the resulting calibration curves and the gravimetric determination of the nanocarrier mass (7.7 mg), the photometric analysis resulted in high drug loads of 28 wt-% ITC and 29 wt-% UMP as well as an ITC : UMP drug ratio of 1 : 1.8.

In sum, the results of total organics combustion with TG, elemental analysis (EA) and photometry are in agreement and result in a good coincidence regarding the composition and drug load of the ITC/TocP@ZrO(UMP) nanocarriers.

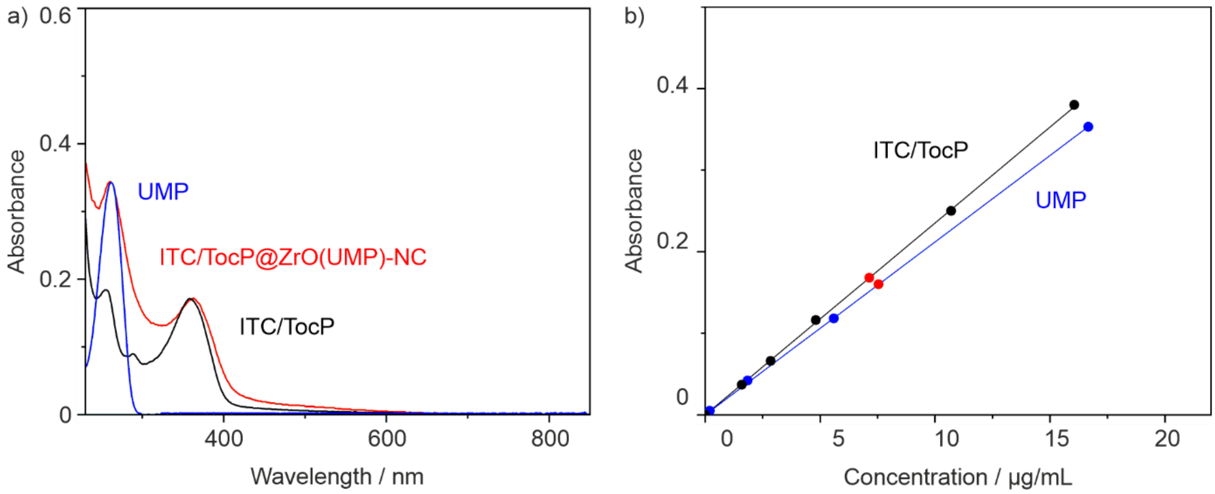

Figure S5: Photometric analysis of the as-prepared ITC/TocP@ZrO(UMP) core@shell nanocarriers: **(a)** UV-VIS spectra of ITC/TocP@ZrO(UMP) core@shell nanocarriers (suspension in water) with the references of TocP-stabilized ITC nanoparticles (suspension in water) and UMP (solution in water); **(b)** calibration curves to extract the ITC and UMP concentrations of the as-prepared ITC/TocP@ZrO(UMP) core@shell nanocarriers (references as black or blue dots; data points of as-prepared nanocarriers as red dots).

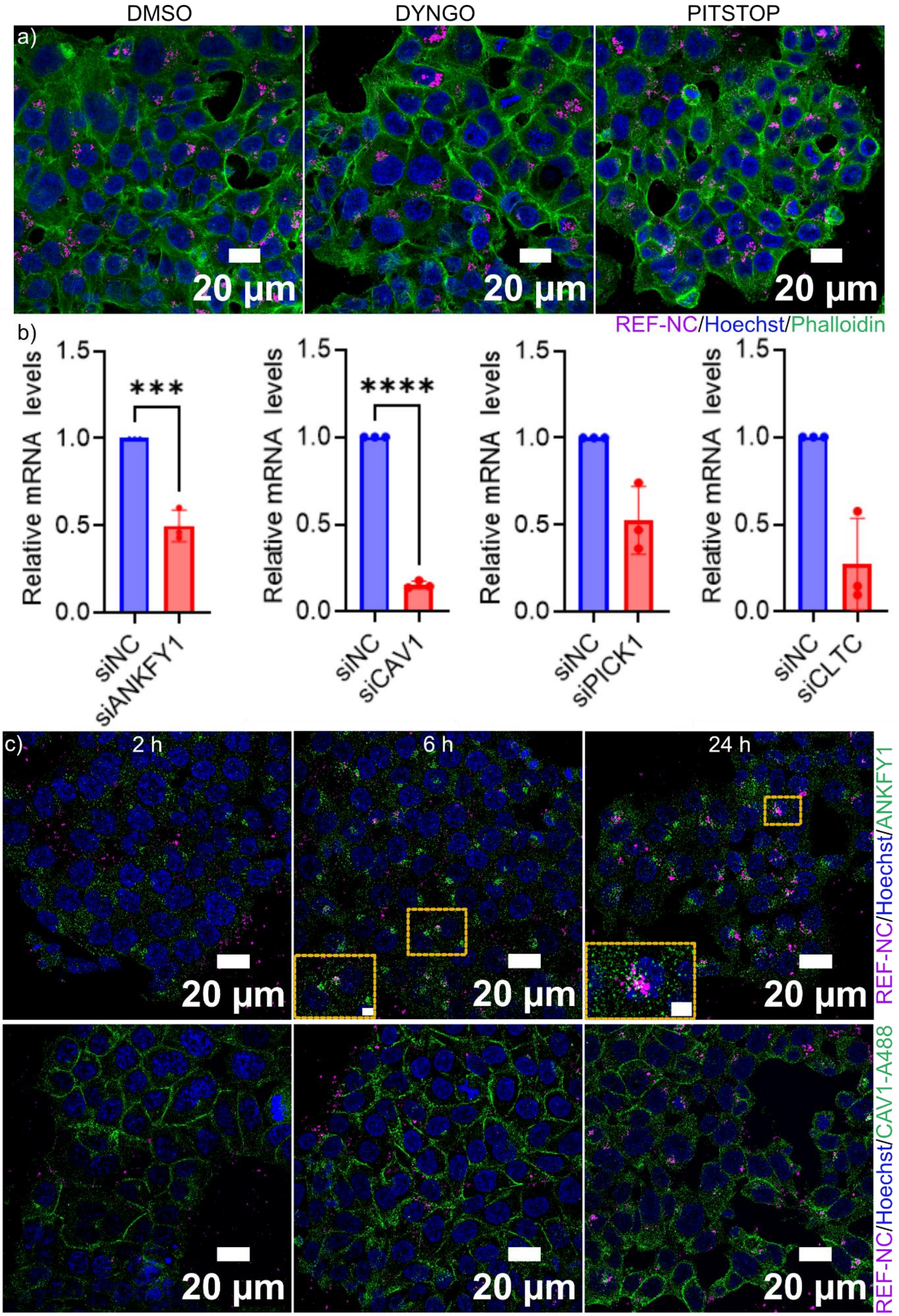

Figure S6: Core@shell nanocarriers are internalized through micropinocytosis. **(a)** Representative confocal images of HCT116 cells pretreated with 10 µM of DYNGO or PITSTOP and then treated with 60.53 µg/mL of DUT647-labeled REF-NC 18 h (magenta), followed by fixing and staining the actin with Phalloidin (green) and nuclei with Hoechst (blue). **(b)** qPCR of Real-time qPCR analysis of shown

mRNA levels for each gene in HCT116 cells transfected with control siRNA or siRNA against the indicated genes for 48 h. Unpaired Student's two-tailed t-test, ***p < 0.001; ****p < 0.0001. Two biological replicates. **(c)** Representative confocal images of HCT116 cells treated with 60.53 µg/mL DUT647-labeled REF-NC for 2 h, 6 h, and 24 h (magenta), followed by followed by immunostaining against ANKFY1 (upper panel) or immunostaining with CAV1 antibody conjugated with AlexaFluor488 (lower panel). Nuclei were stained with Hoechst (blue). The scale bars in the zoomed insets of (c) represent 5 µm.

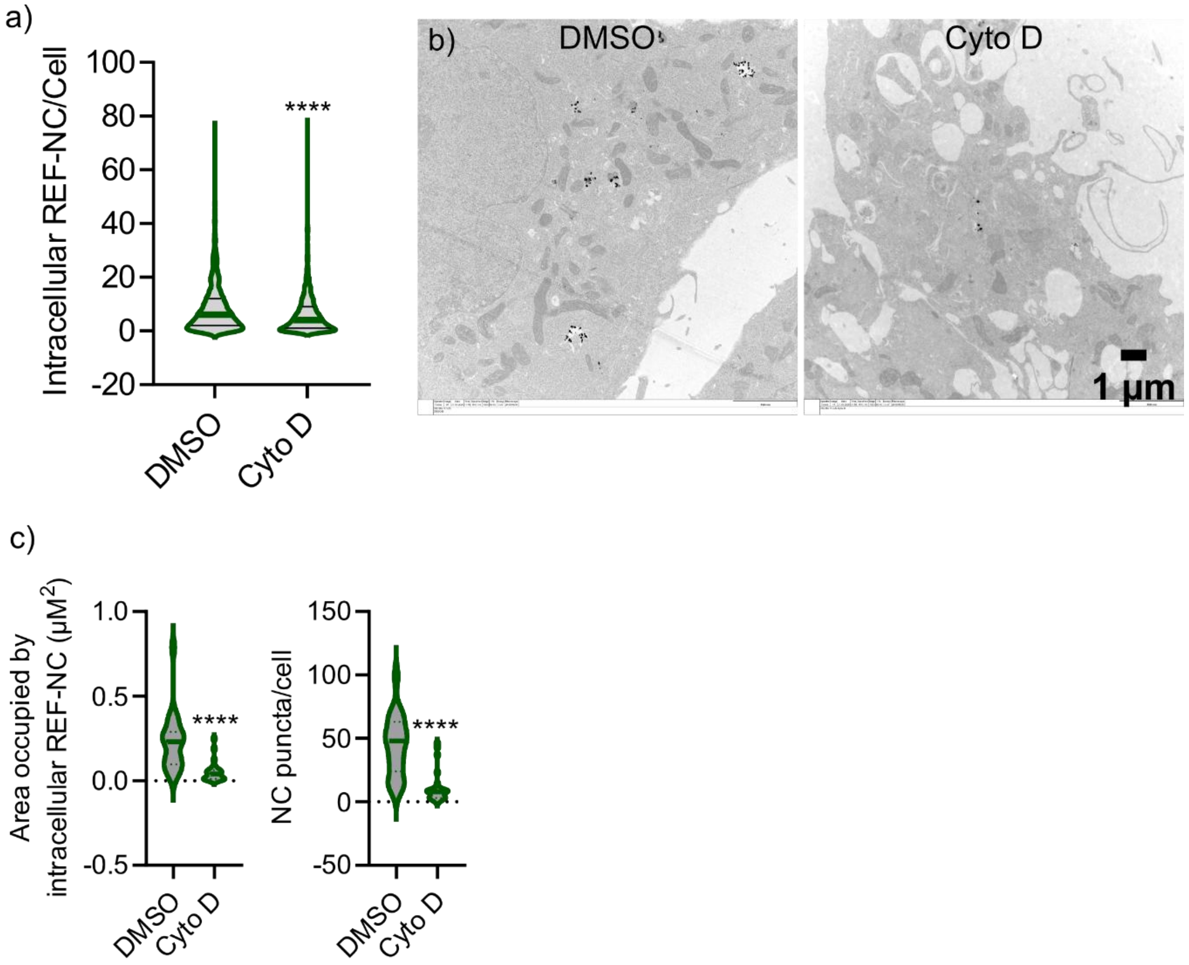

Figure S7: Core@shell nanocarriers are internalized through micropinocytosis. **(a)** Quantification of intracellular REF-NC per cell from (Figure 4d), analyzed using CellProlifer. Data are from three biological replicates; ****p < 0.00001, (unpaired two-tailed Student's t-test). **(b)** Representative electron microscopy images of HCT116 cells pretreated with DMSO or cytochalasin D (Cyto D) for 2 hours, followed by treatment with 60.53 µg/mL REF-NC for 24 hours. **(c)** Box plots showing quantification of EM images from (b), comparing DMSO- and Cyto D-treated HCT116 cells. Left: intracellular area occupied by REF-NC. Right: number of intracellular REF-NC puncta per cell.

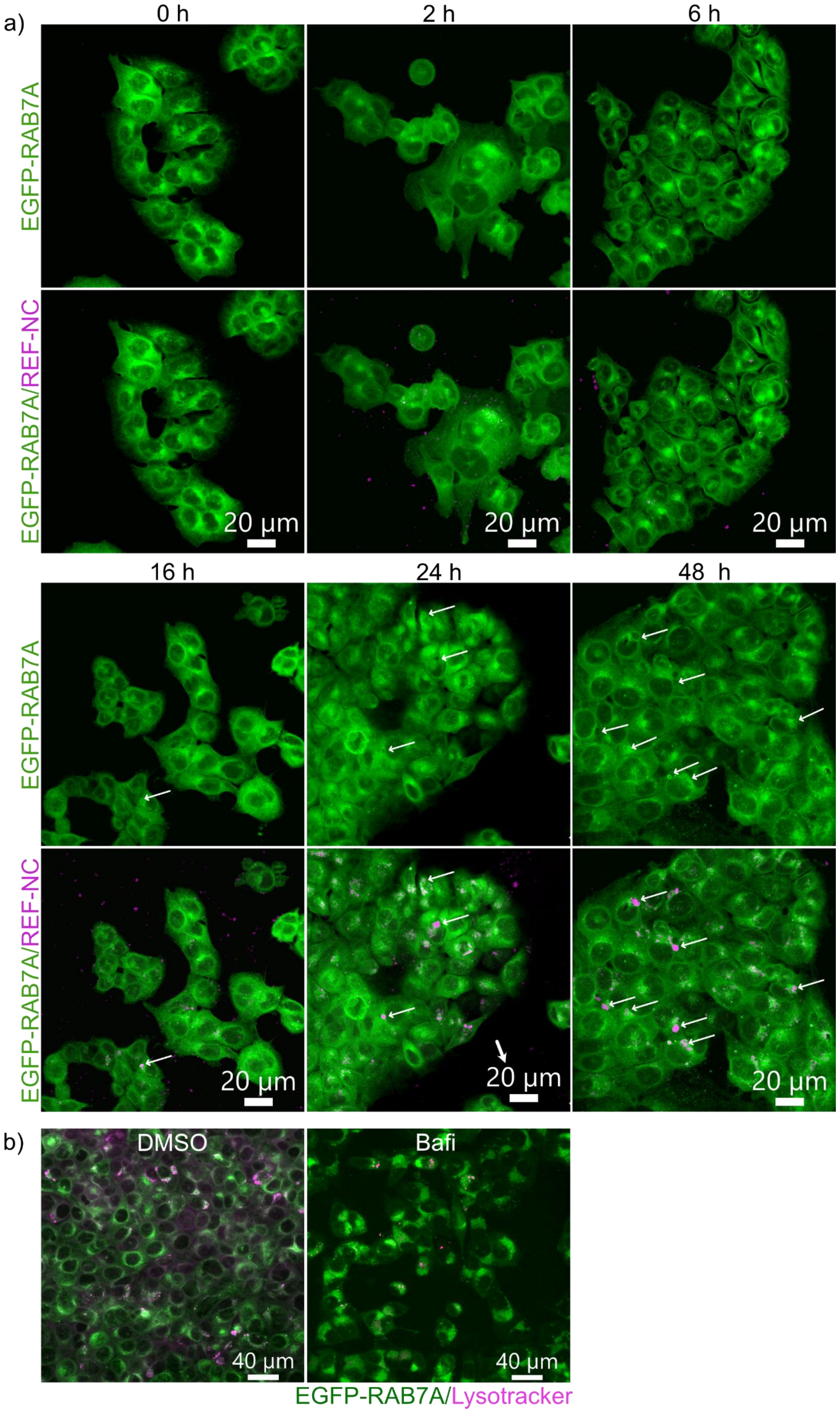

a)
0 h
2 h
6 h
16 h
24 h
48 h
EGFP-RAB7A
EGFP-RAB7A/REF-NC
20 µm
b)
DMSO
Bafi
40 µm
EGFP-RAB7A/Lysotracker

Figure S8: Core@shell nanocarriers undergo intracellular endosomal trafficking. **(a)** Representative confocal microscopy images of HCT116 cells stably-expressing EGFG-RAB7A (green), untreated (0 h) or treated with 60.53 µg/mL DUT647-labeled REF-NC (magenta) at different time points 2 h, 6 h, 16 h, 24 h, and 24 h. The white arrows indicate nanocarrier signals in Rab7-positive vesicles **(b)** Representative spinning disk confocal microscopy images of HCT116 cells stably-expressing EGFP-RAB7A (green) treated with 200 nM bafilomycin A1 (Bafi) for 18 h to inhibit endolysosomal acidification and then stained with 100 nM of Lysotracker DND-99 (green) before fixation and imaging.
